# Data-Efficient Distal Engineering of Fluorinase Using Zero-Shot Models

**DOI:** 10.1021/cbe.6c00028

**Published:** 2026-08-05

**Authors:** David Harding-Larsen, Brianna M. Lax, Christopher Kurt Weingarten, Aboubakar Sako, Stanislav Mazurenko, Ditte Hededam Welner

**Affiliations:** † The Novo Nordisk Foundation Center for Biosustainability, 5205 Technical University of Denmark, Søltofts Plads 220, 2200 Kongens Lyngby, Denmark; ‡ Loschmidt Laboratories, Department of Experimental Biology and RECETOX, Faculty of Science, 37748 Masaryk University, Kamenice 5, 625 00 Brno, Czech Republic; § International Clinical Research Center, St. Anne’s University Hospital, Pekařská 664/53, 602 00 Brno, Czech Republic

**Keywords:** biocatalysis, biofluorination, distal mutagenesis, zero-shot, low-N modeling, enzyme engineering

## Abstract

Fluorinases have high potential for industrial biofluorination, but any applications have been precluded by low catalytic efficiency and resistance to active site engineering. In this work, we employed PRIZM, a computational workflow utilizing an existing low-N data set and zero-shot models for *in silico* prediction of activity-enhancing mutations at distal sites. The combination of these predictions with expert opinion led to the identification of 21 fluorinase mutants with enhanced relative activities, while 3 variants showed increased melting temperatures. A mutation in the hexameric interface, K237R, resulted in the largest stability gain, a more than 3.2-fold improvement in catalytic efficiency at 57 °C, and an 8-fold increase in relative activity at 62 °C. These results highlight the potential of distal fluorinase engineering for improving properties required to realize its industrial applications.

## Graphical abstract

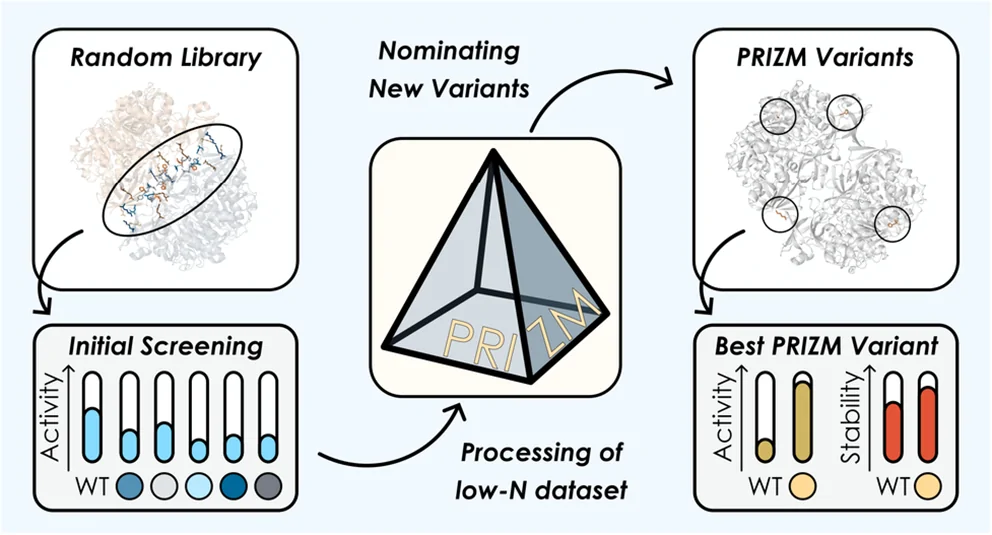

## Introduction

Fluorinases (5′-fluoro-5′-deoxyadenosine synthases, EC 2.5.1.63) are the only known class of enzymes able to directly catalyze carbon–fluorine bond formation, making them the subject of great interest for potential industrial bioproduction of organofluorides, which currently make up over 50% of agrochemicals,^1,2^ 20% of marketed pharmaceuticals,^3,4^ and contribute to many other sectors.^5,6^ Fluorinases perform bimolecular nucleophilic substitution (S_N_2) on *S*-adenosyl-l-methionine (SAM) with fluoride as the nucleophile, producing 5′-fluoro-5′-deoxyadenosine (FDA) and l-methionine^7,8^ (Scheme 1). There are currently 18 functionally characterized fluorinases,^9,10^ and two crystal structures available (fluorinases from *Streptomyces cattleya* and *Streptomyces* strain sp. MA37).^11,12^

**Scheme 1:**
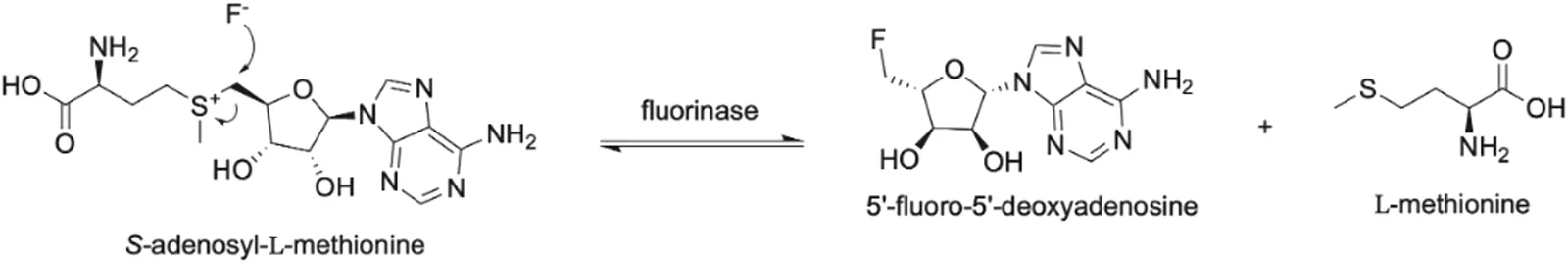
Reaction Scheme of Fluorinase-Mediated SAM Conversion into FDA and l-Methionine

Despite their potential, the fluorinases are infamous for their prohibitively slow turnover rates (*k*_cat_ = 0.16–0.41 min^–1^) and narrow substrate scopes that have precluded any industrial implementation.^9^ Additionally, fluorinases have been historically resistant to catalytic improvements from active site mutagenesis, with previous active site engineering campaigns affording only incremental improvements.^13,14^ One recent study performed rational engineering of the fluorinase at allosteric sites, and when combined with reaction medium optimization, was able to improve the turnover rate by 50×, representing a landmark improvement in biofluorination.^15^ Thus, the existing literature suggests that the efficiency of fluorinases may perhaps be best enhanced outside of the active site.

Fluorinases belong to the “SAM-Dependent Hydrolases/Halogenases” superfamily, and despite similar monomeric structures to other members, they represent the only known hexameric halogenases.^12,16^ The homohexameric structure is a dimer of trimers (D3 symmetry), containing a total of six identical monomers and six active sites. The monomer contains two domains, which are connected by a long loop present in all known fluorinases except for the archaeal fluorinase from *Methanosaeta* sp. PtaU1.Bin055.^9^ The active sites are formed at each of the monomer interfaces in the trimers, containing residues from the N-terminal domain of one monomer and residues from the C-terminal domain of the other, obviating the need for trimerization in catalysis.^12^ Our group recently showed that hexamerization, however, is not required for catalysis, although it does confer reaction efficiency by decreasing the Michaelis constant (*K*_m_) for SAM by 2 orders of magnitude.^17^ The discovery of this functional benefit of hexamerization uncovered the hexameric interface as an interesting and unexplored target for engineering.

In recent years, protein engineering has seen the emergence of machine learning (ML) as a promising tool for guiding the mutagenesis process.^18–20^ However, the performance of supervised models is tied to both the quantity and quality of data sets used for model training–properties that are often inversely related. An alternative approach is to employ these foundation models as zero-shot predictors, where the model outputs are used as proxies for fitness and activity changes caused by mutagenesis.^21^ Since they do not rely on supervised learning, zero-shot predictors are versatile and simple to employ. Zero-shot predictors can be used across different scales–from engineering individual proteins^22–25^ to predicting variant effects at the proteome,^26^ genome,^27^ or even metagenome level^28^–highlighting the versatility and broad biological knowledge contained in these pretrained models.

In this study, we aimed to utilize ML to explore the distal mutational landscape in an intentional and efficient manner. First, we generated a low-N variant activity data set of the fluorinase from *Streptomyces* sp. MA37 (hereafter referred to as “FlA”). We then employed our Protein Ranking using Informed Zero-shot Modelling (PRIZM) workflow, a two-phase pipeline, for identifying protein variants with enhanced properties.^29^

We expressed and characterized the best predicted single mutants, with ∼28% having higher activity than WT. We also explored combinations of mutations as well as semirational engineering informed by the ranked *in silico* library, both of which were successful. The best mutant K237R demonstrated a ∼3.6-fold improvement in catalytic efficiency at 57 °C, as well as a ∼2.4 °C increase in an apparent melting temperature (Δ*T*_m,app_).

Overall, this study shows that distal regions impact the activity of FlA and highlights the benefit of using ML to navigate these large sequence spaces. It also demonstrates the utility of zero-shot learning in engineering challenging, poorly understood enzymes such as FlA.

## Results and Discussion

### Initial Experimental Characterization

We began our exploration of the FlA sequence space by generating a combinatorial mutant library of the hexameric interface residues. We targeted the hexameric interface for our mutational campaign since it is a unique feature of FlA within the halogenases and due to the limited success with active site engineering. Since the active sites are located at the monomer interfaces within the trimers, there is no overlap in residues that make up the active sites and those that participate in the hexameric interface. Based on our previous work, which demonstrated the hexameric region as a modulator of FlA activity,^17^ we selected seven residues that facilitate this dimerization for our mutagenesis campaign (Figure 1A,B). The resulting library consisted of 1.28 billion variants, each containing mutations at between one and seven of these residues.

**Figure 1:**
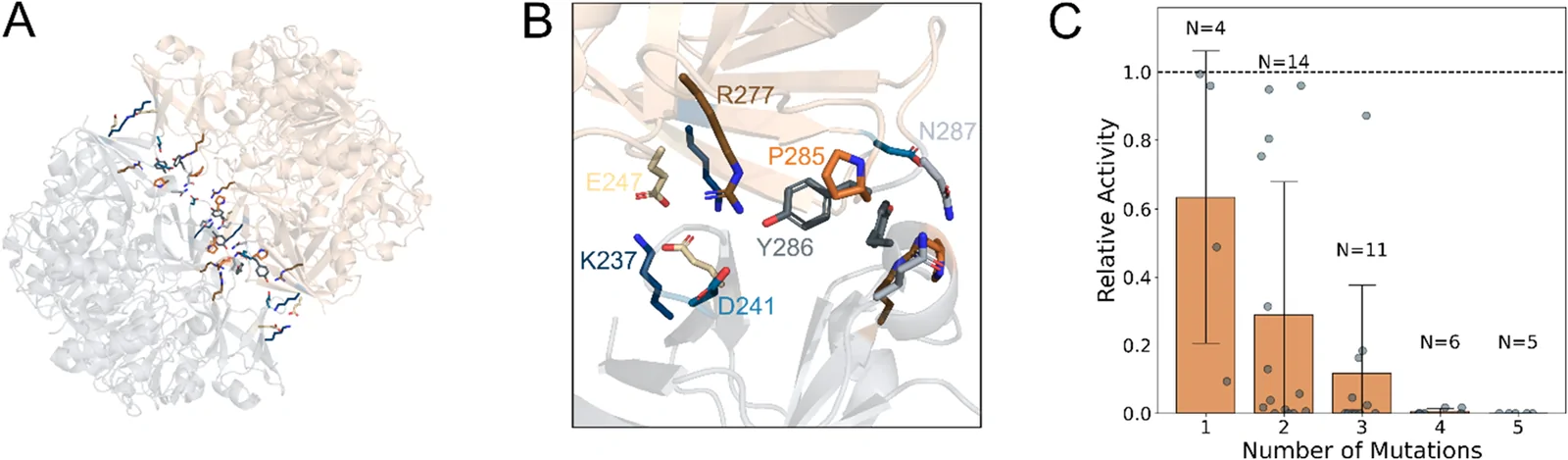
Initial FlA experimental examination. (A) Structural cartoon representation of the hexameric FlA (PDB ID: 5B6I), with each trimer distinctly colored and the six sets of seven hexameric interface residues shown as sticks. (B) Zoom-in on one of the contact points of the hexameric interface. (C) Relative activities for FlA variants in the low-N data set as a function of the number of mutations per variant. Orange bars show average relative activity, gray circles indicate individual measurements, and error bars denote the standard deviation. The dashed line denotes WT activity of 1, with all variants being less active than WT.

Without a high-throughput screen available, it was unfeasible to characterize the entire library, so we randomly chose a small sample for initial characterization to look for trends between the mutation sites. Although we previously showed that hexamerization was not necessary for activity, the trimeric mutants that were tested contained only 1–2 mutations in the hexameric interface.^17^ In this study, we characterized 40 variants with one to five mutations (Figure S1, Table S1). We observed an inverse relationship between the number of mutations per variant and relative activity, with the majority of variants with four or more mutations losing all activity (Figure 1C). Moreover, none of the tested variants had higher activity than WT, which prompted us to use ML to explore the mutational landscape *in silico* and narrow down promising variants for the expression and characterization in the lab.

### PRIZM Processing of FlA

Given the success of the PRIZM workflow at identifying beneficial nonactive site mutants from small data sets in our previous work,^29^ we decided to implement the workflow on our hexameric interface data set. First, we used the 40 characterized variants to identify the best model for describing FlA relative activity (Figures 2A–C and S2). ESM-IF1,^30^ a structure-based zero-shot model, exhibited the best performance overall, while ESM-1b^27,31^ was the best performing sequence-based model, and TranceptEVE^22^ was the best performing model utilizing MSA information. We observed a significant difference in model performance between the best and worst models for our FlA data set (Figure 2C), reinforcing that PRIZM can distinguish between the different zero-shot models using low-N data sets. While the ESM-IF1 scores were unable to accurately model the experimental data, a general trend was observed, where higher ESM-IF1 scores were associated with higher experimental values, in contrast to the low-scoring model UniRep. Notably, variants with no activity were very poorly predicted by both models, with inactive mutants giving rise to the full spectrum of scores. Comparing the ESM variants also highlighted that newer models do not always equal better performance, as the older ESM-1b outperformed the newer ESM-2 ^28^ (Figure S2).

**Figure 2:**
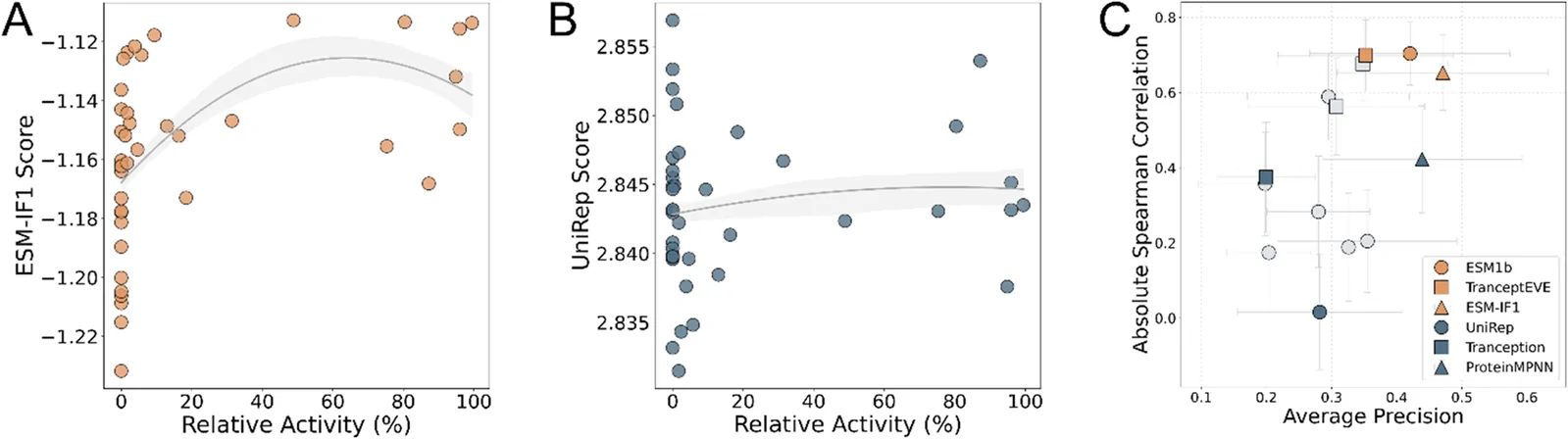
PRIZM model selection for FlA data set. (A, B) Correlation between the experimental relative activities of the FlA low-N data set and either ESM-IF1 scores (A) or UniRep scores (B). A second order polynomial is fitted to the data and is only used to guide the eye (the gray line denotes the regression estimate and the shaded area shows the confidence interval calculated using bootstrapping). (C) Spearman correlation and average precision for zero-shot scores and experimental values for the different zero-shot models of PRIZM (circle = sequence-based model, square = MSA-based model, and triangle = structure-based model; blue and orange denote the highest and lowest-correlated models for each model category, respectively, as identified by PRIZM). Error bars indicate the standard deviation estimated from bootstrapping the data set (*N* = 1000).

We then constructed a full *in silico* FlA single-point mutant library and processed it using the top performers of each predictor category: ESM-1b (sequence-based), TranceptEVE (MSA-based), and ESM-IF1 (structure-based). This was partly due to the similar absolute Spearman correlations of the three models, but also because this allowed us to probe the effect of using sequence, evolutionary, and structural information for prediction. Using the ranked *in silico* library, we employed a *top N* approach for selecting the ten highest-ranked mutants for each zero-shot model. Furthermore, while the model scores were not highly correlated, we found several mutants that were predicted to be better than WT by two of the models (Figures S3–S4), suggesting that these variants are consistently favored by different zero-shot models trained on distinct types of protein information. As shown previously, combining model predictions from PRIZM can lead to enhanced variants.^29^ We therefore also selected variants from model pairings–either by including all mutants predicted to be better than WT, or, when more than ten such mutants were identified, by combining the rankings from both zero-shot models and selecting the top candidates based on this combined ranking. Interestingly, no mutant scores were predicted to be better than WT by all three models at the same time. In total, 42 unique single mutants were chosen for experimental validation and named “the PRIZM variants” (Table S2).

Lastly, we also conducted PRIZM evaluations of all FlA double mutants, though we observed an almost perfectly linear relationship between double mutant scores and the sum of their corresponding single mutant scores (Pearson correlation >0.99 for all models, Figure S5). This is consistent with previous studies observing that zero-shot predictors struggle to predict epistatic effects.^32^

### Experimental Validation of PRIZM Variants

We expressed the PRIZM variants recombinantly in *Escherichia coli* and purified them using affinity chromatography. Two variants (P165N and F65I) did not express solubly and were omitted. For the remaining 40 variants, we examined the relative activity after 2 min (Figure S6) and 3 h (Figure S7). Across all three top-performing zero-shot models, the activities of the selected variants were shifted toward higher values relative to the original low-N data set, with many variants reaching or exceeding WT activity (Figure 3A). Interestingly, we observed significant variability in the distributions of the three selected zero-shot models; the variants chosen using ESM-1b scores were all worse than WT for both time points, while the variants of the structure-based ESM-IF1 had a large spread of activities, from almost no activity to higher than WT. The TranceptEVE variants performed the best on average, with the majority exhibiting enhanced activity compared to WT. These trends were mirrored in the variants selected by pairs of models, as the variants with ESM-1b being one of the selection models performed worse than those selected by TranceptEVE and ESM-IF1. The latter combination resulted in the best collection of variants, with a median relative activity of 1.085.

**Figure 3:**
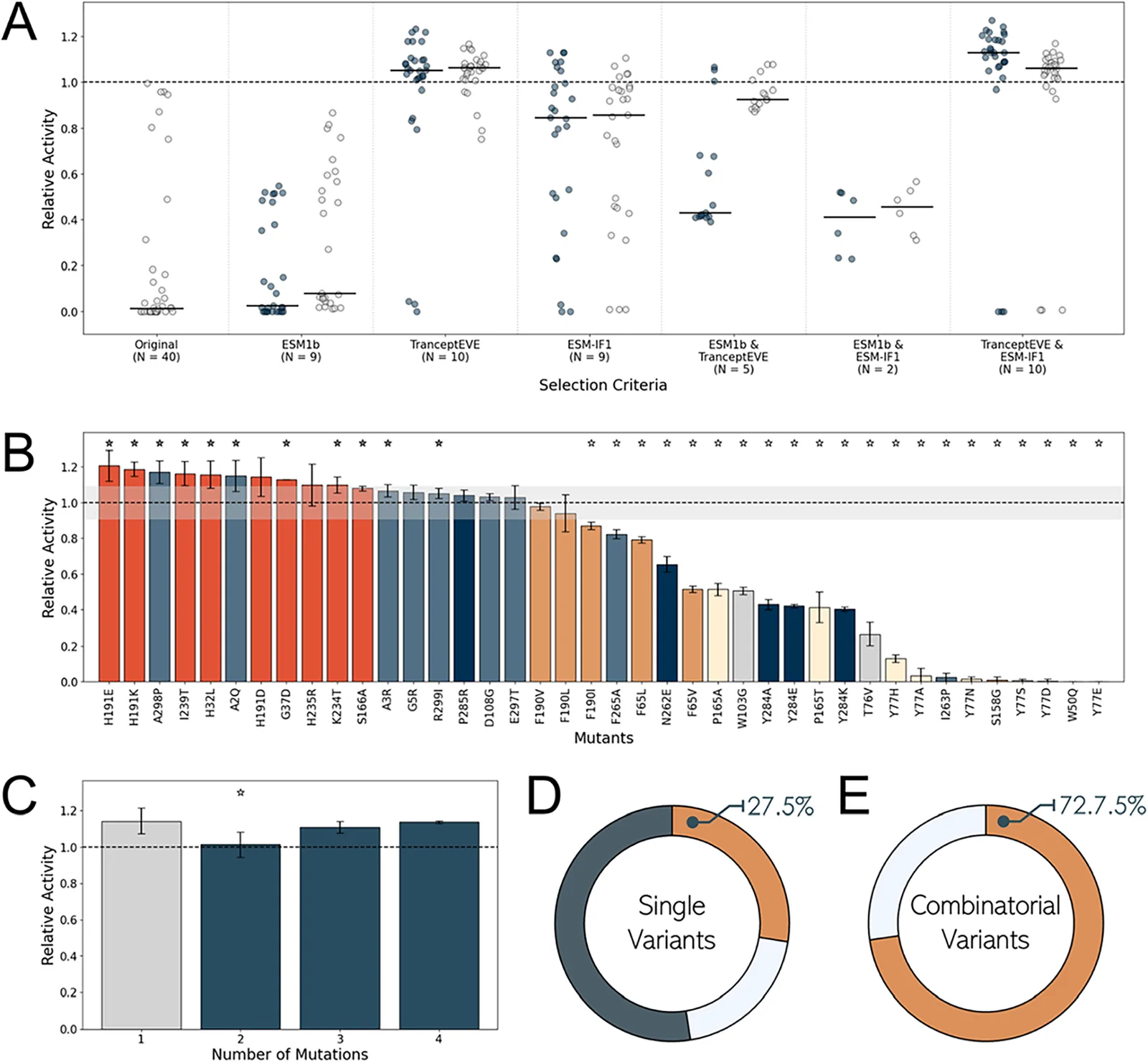
Relative activity of PRIZM mutants. Dashed lines denote the WT activity. (A) Point clouds of relative activities after 2 min (blue) and 3 h (gray) for variants selected using different criteria. “Original” denotes the original low-N FlA data set. Horizontal lines denote median. (B) Relative activities after 2 min of single mutants from different selection criteria: beige = ESM-1b, light-blue = TranceptEVE, orange = ESM-IF1, gray = combination of ESM-1b and ESM-IF1, dark-blue = combination of ESM-1b and TranceptEVE, and red = combination of TranceptEVE and ESM-IF1. Stars denote variants with relative activity at 2 min significantly different from WT (one-sided Welch test with α = 0.05, gray = better than WT, white = worse than WT). Although certain variants are selected based on multiple selection criteria, each is displayed only once in this figure (for a comprehensive overview of all selections, refer to Figure S4). Error bars indicate the standard deviation of biological replicates. (C) Average relative activities after 2 min as a function of the number of mutations per variant. Gray = selected single variants, blue = combinatorial variants. Error bars indicate the standard deviation, while the relative activities of individual combinatorial mutants can be found in Figure S11. Error bars indicate the standard deviation of biological replicates. Stars denote combinatorial variants significantly different from single variants (one-sided Welch test with α = 0.05). (D, E) Percentage of variants with significantly higher activity than WT (orange), WT-like activity (light blue), or lower activity than WT (dark gray) at 2 min for PRIZM/single (D) and combinatorial (E) variants. Significance was determined using a one-sided Welch test (α = 0.05).

Interestingly, such trends were also observed for the previously described model selection step of PRIZM. The purely sequence-based models generally performed worse than both MSA-based or structure-based models, with only ESM-2 and ESM-1b being competitive among the sequence models (Figure S2). Furthermore, the difference in performance between the original purely sequence-based UniRep model and the evotuned variant that also utilized MSA information highlights the importance of evolutionary patterns for understanding FlA activity. Together, these findings suggest that sequence alone might not be enough to guide engineering of FlA activity, and that epistatic effects captured in structural information and evolutionary conservation could play a significant role in this enzyme.

Interestingly, the relative activities converged around the WT value at the 3 h data point (Figure S8). Since product inhibition is a known limitation of fluorinases catalysis,^15,33^ we hypothesized that differences in initial rate are diminished at the 3 h time point due to production of FDA. To test this, we performed a time-course assay in the presence of 0, 25, or 50 μM FDA added at the beginning of the reaction and monitored both FDA and SAM concentrations (Figures S9–S10). We observed that the addition of FDA at the beginning of the reaction decreased the amount of FDA produced, and that more than 50% of the SAM remained, attributing this relative activity convergence to product inhibition. Thus, we continued with the 2 min data as the basis for identifying interesting mutations, due to the better separation of best and worst variants.

We observed 11 variants with activities significantly above WT (Figure 3B), with the best variant, H191E, having a relative activity of 120.7%. To explore potential epistasis, from these 11 variants, we selected four mutations for combinatorial analysis: H191E, H32L, G37D, and S166A. Importantly, we decided against using the zero-shot scores to nominate combinatorial mutants, given the almost perfectly linear correlation between double mutant scores and the sum of the corresponding single mutant scores described above (Figure S5). These variants were selected since they were the highest-ranked variants with unique positions from the top-performing PRIZM result (combination of TranceptEVE and ESM-IF1, Table S2). Interestingly, S166A was previously reported to be important for enzyme activity for the fluorinase from *Amycolatopsis* sp. CA-128772 due to its influence on the fluoride ion channel.^14^ This study analyzed 62 mutants to identify this mutation, whereas it was ranked as the tenth best by ESM-IF1 and third best by the combination of TranceptEVE and ESM-IF1, significantly reducing the number of variants tested to arrive at this interesting mutation. Lastly, it is worth noting that all the active site mutations predicted to be better than WT exhibited little activity, with an average relative activity of 2.5%, compared to an average relative activity of 85.7% for distal mutations, confirming the challenge of engineering the active site of fluorinases.^12^

As we previously observed that the double mutants’ zero-shot scores were essentially the same as summing the individual single mutants, we instead used the four nominate combinatorial variants. We combined these four single mutants into double, triple, and quadruple mutants, denoted as “the combinatorial variants” (Figures 3C and S11). Here, most of the double mutants exhibited no enhanced performance compared to WT, with only H32L-H191E having similar performance to the corresponding single mutant variants. The triple and quadruple mutants recovered the activity levels of the single mutant variants but did not exhibit additional synergy. Interestingly, we observed a less pronounced version of the same trend as the original low-N data set, where an increase in the number of mutations was inversely correlated with the relative activity of FlA (Figure 1C). In the original low-N data set, the mutations are close together in the hexameric interface, and thus the residues can more directly interact with each other. This proximity creates stronger, more incompatible changes in the structure that have large effects on activity. However, since the PRIZM mutations are not as close together, their indirect interaction through epistatic effects, rather than direct interaction between the residues, is driving the activity of the combinatorial mutant. In both cases, we observe that the combination of mutations generates destructive interference, rather than additive or synergistic effects, but to different degrees of severity.

In conclusion, combining PRIZM with a greedy approach resulted in a best-performing single mutant (H191E) with 120.7% relative activity. H191 is located within the linker connecting the two domains of the FlA monomer (Figure S12), a site that is spatially distant from conventionally studied regions. As such, H191E would likely have been overlooked in traditional rational engineering approaches. This finding illustrates the utility of zero-shot learning methods in identifying functionally relevant mutations in regions of the protein landscape that lack prior functional annotation or intuitive significance. Overall, we had a success rate for variants with higher activity than WT of 27.5% (11/40) for the PRIZM/single mutants and 50% (3/6) for the double mutants, while all the triple and quadruple mutants had activity above the WT, resulting in an overall 72.7% success rate for the combinatorial variants (Figure 3D,E).

### Targeted Engineering Using PRIZM and MSA

Following our PRIZM and combinatorial variants, we tested a more targeted engineering approach, hypothesizing that combining expert knowledge with zero-shot prediction would enhance success rate and variant performance; Our experimentally validated PRIZM variants with enhanced activity located primarily to three regions of interest (Figure 4A). The first is the hexameric interface, *i.e*., the original target of our low-N data set. The two others are the N-terminal tunnel and the area around the active site (henceforth denoted as the second-shell of the active site). We utilized the PRIZM rankings to identify residues within these regions predicted to be better than WT by both TranceptEVE and ESM-IF1. To complement the PRIZM analysis, we constructed an MSA of previously studied fluorinase homologues^9^ (Table S3). This homologue MSA was utilized to select mutations at our identified positions that were either conserved across multiple fluorinases (consensus) or present in the faster fluorinase homologues.

**Figure 4:**
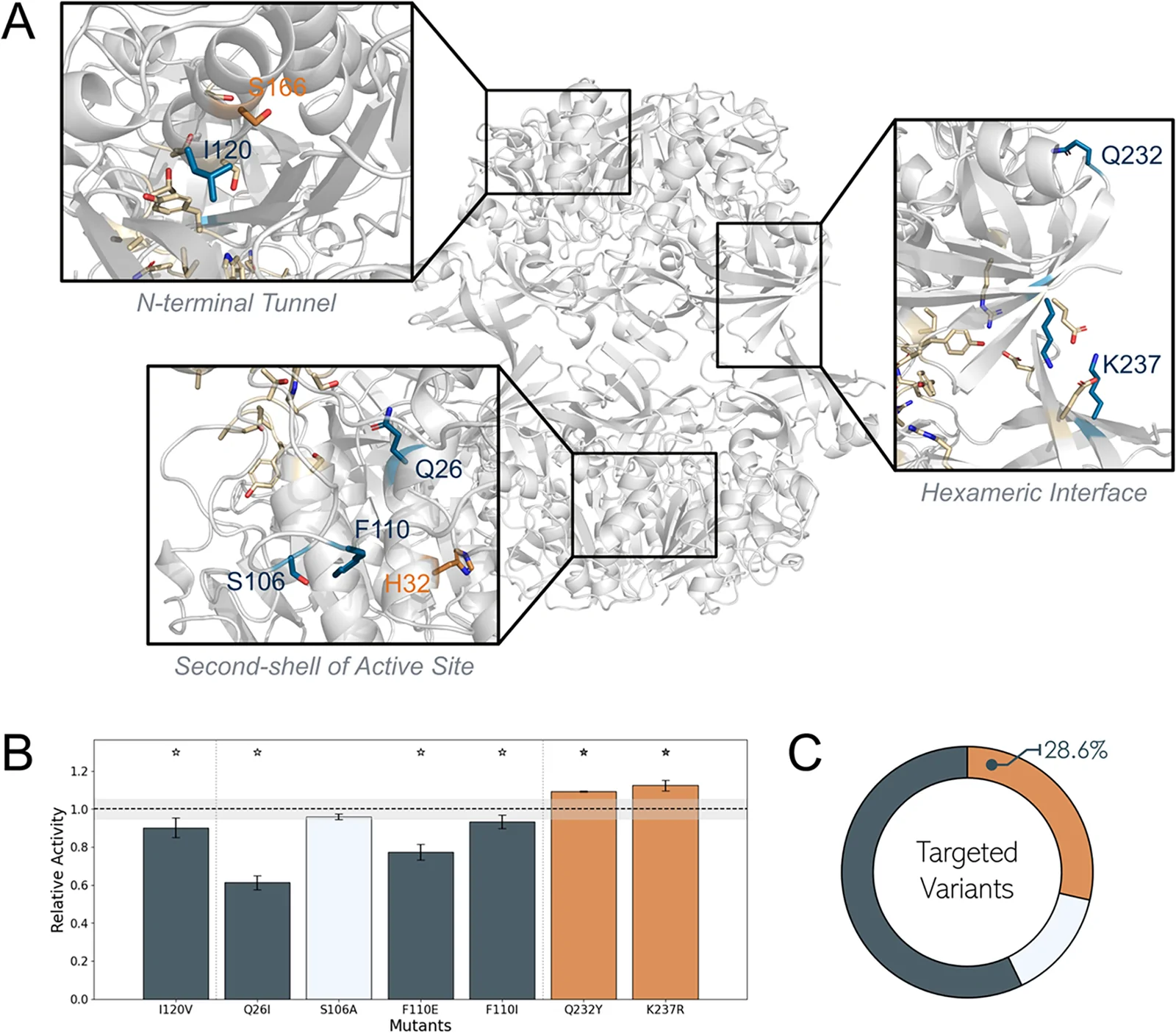
Targeted engineering of FlA. (A) Close-up of regions of interest used for targeted engineering, with residues of note shown as sticks (PDB ID: 5B6I). Newly selected residues are colored blue, previous PRIZM variants are colored orange, while active site residues or initial hexameric interface residues are colored beige. (B) Relative activity of targeted variants at 2 min. Variants with significantly higher activity than WT are colored orange, WT-like variants are colored light blue, while those with lower activity than WT are dark gray. Dashed line denotes the WT value of 1. Error bars indicate the standard deviation of biological replicates with *N* = 3. Stars denote variants with relative activity at 2 min significantly different from WT (one-sided Welch test with α = 0.05, gray = better than WT, white = worse than WT) are denoted by a star. (C) Percentage of variants with significantly higher activity than WT (orange), WT-like activity (light blue), or lower activity than WT (dark gray) at 2 min for targeted variants. Significance was determined using a one-sided Welch test (α = 0.05).

In the hexameric interface, we identified Q232Y and K237R, with both mutations being highly conserved across the other fluorinase homologues. For the N-terminal tunnel, we selected I120V, as the MSA showed the valine to be present at this position in several of the homologues, including two of the faster fluorinases. Lastly, four mutations were chosen at the second shell of the active site: Q26I, with the isoleucine being present in the fastest homologue, F110I and F110E, where the mutated residue was present across multiple homologues, as well as S106A. In total, we selected and characterized seven additional mutations, denoted as the “targeted variants”.

Many of these targeted variants showed either close to or worse activity than that of the WT enzyme (Figure 4B). Especially the Q26I variant, situated close to the active site, showed decreased performance, consistent with previous challenges of improving the enzyme with mutations near the active site. However, both mutations in the hexameric interface exhibit significantly enhanced performance, confirming our initial hypothesis of the importance of this region for FlA activity. Overall, our targeted approach achieved a success rate of 28.6% (2/7), still outperforming our initial greedy approach and highlighting strength of combining expert knowledge with the versatility and power of the raw zero-shot predictions (Figure 4C).

### Biochemical Characterization of Top Variants

We then investigated the underlying causes of the increased relative activities in our top variants: the four previously selected PRIZM variants, H32L, G37D, S166A, and H191E, as well as the best targeted mutant, K237R.

We first quantified the *T*_m,app_ of each of these variants using differential scanning fluorimetry (DSF), in reaction buffer alone, with SAM, or with F^–^ (Figures 5A and S13). We observed that the *T*_m,app_ values of H191E, H32L, and K237R were all increased compared to WT in all three conditions, with the latter having a Δ*T*_m,app_ value of up to ∼2.4 °C in the presence of SAM. In particular, K237R exhibited an increase in Δ*T*_m,app_ in the presence of the substrates, suggesting a synergy between the mutation and the interaction of the protein with these molecules. We see that all mutants except S166A and all except G37D and S166A have larger increases in Δ*T*_m,app_ after the binding of F^–^ and SAM, respectively, compared to WT (Figure S13).

**Figure 5:**
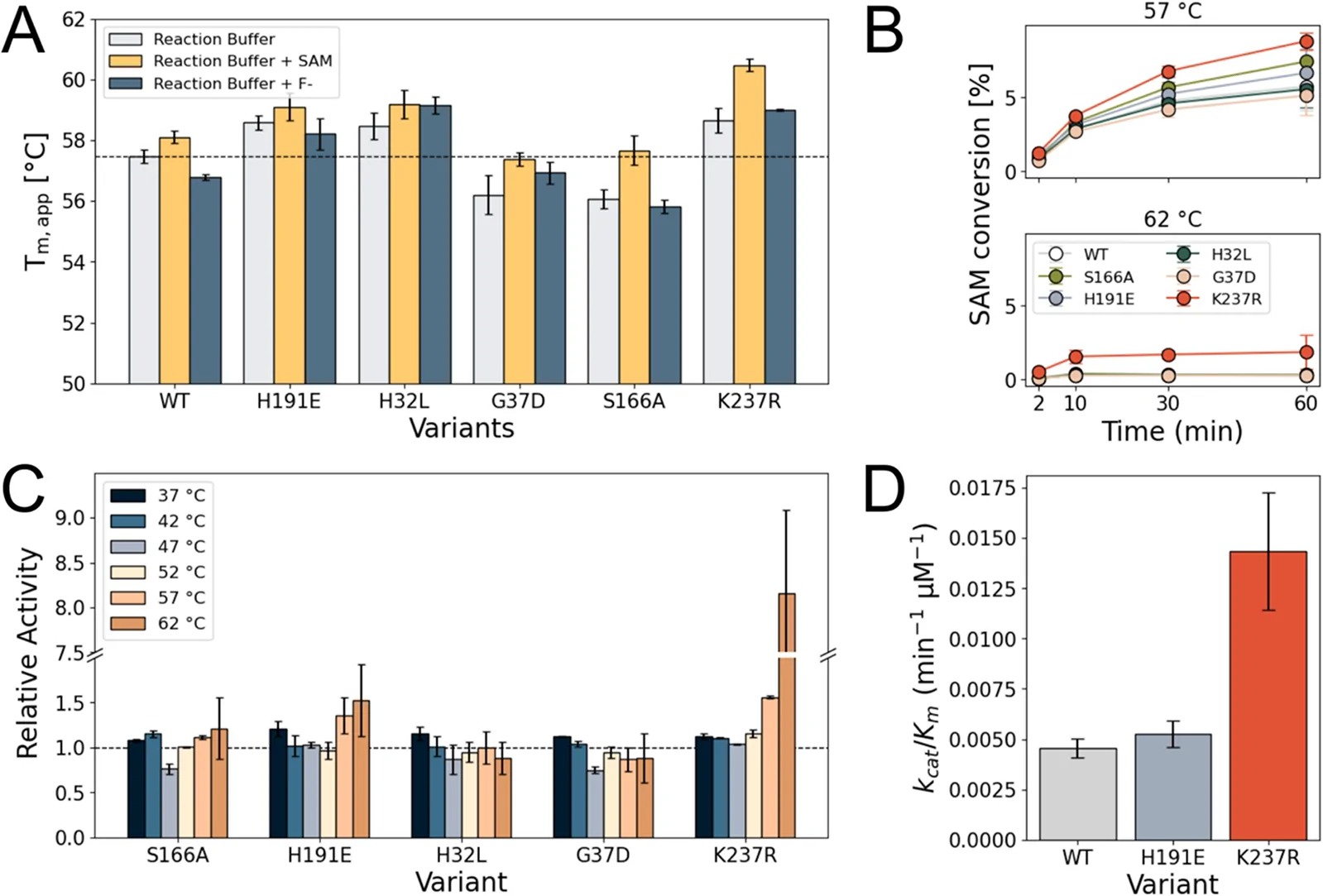
Biochemical characterization of FlA WT and top FlA variants. Error bars indicate the standard deviation of biological replicates with *N* = 3. (A) *T*_m,app_ in reaction buffer alone (gray), with SAM (yellow), or with F^–^ (blue). The dashed line indicates the *T*_m,app_ of WT in the reaction buffer. (B) SAM conversion at 57 °C (top) and 62 °C (bottom) at 2, 10, 30, and 60 min. (C) Relative activity after 2 min at different temperatures, with the dashed lined indicating a relative activity of 1. (D) Catalytic efficiency at 57 °C.

To complement our thermostability assay, we examined the activity of the variants at 2, 10, 30, and 60 min at temperatures ranging from 37 to 62 °C (Figures 5B and S14–S15). We observed an optimal temperature of WT around 52 °C, with the enzyme appearing mostly inactive at 62 °C, which aligns with this being above the enzyme’s *T*_m,app_. Despite the increases in *T*_m,app_ values for many of the mutants, the optimal temperature remained around 52 °C for all variants. Interestingly, while S166A, H191E, and K237R demonstrated enhanced relative activity at 37 °C, they outperformed WT to a much greater extent at 57 and 62 °C (Figures 5C and S16). The three variants achieved a 21.4, 52.6, and 716.2% increase in relative activity, respectively, at 62 °C. Given these impressive increases in relative activities for H191E and K237R at elevated temperatures, we performed Michaelis–Menten kinetic analysis at 57 °C (Figures 5D, S17, and Table 1). We did not observe an increase in the turnover rate of either of these variants, but rather, an improvement in catalytic efficiency related to decreased Michaelis constants (*K*_m_) for SAM. The *K*_m_ values for H191E and K237R were ∼1.2-fold and ∼3.6-fold lower than WT, respectively, leading to 18% and ∼218% increases in catalytic efficiency. For K237R, this is in alignment with our data showing that the greatest stability benefits from these mutations are observed in the presence of SAM. We believe that these mutations are able to improve SAM binding, which then improves the stability of the protein, synergistically improving the efficiency of the enzyme without affecting the turnover rate.

**Table 1:** Biochemical Characterization of WT, H191E, and K237R^a^

variant	*T*_m,app_ (alone)°C	*T*_m,app_ (SAM)°C	*T*_m,app_ (F^–^)°C	*k*_cat_ min^–1^	*K*_m_ μM	catalytic efficiency min^–1^ μM^–1^
WT	57.5 ± 0.2	58.1 ± 0.2	56.8 ± 0.1	1.10 ± 0.08	244 ± 23	0.0045 ± 0.0003
H191E	58.6 ± 0.2	59.1 ± 0.5	58.2 ± 0.5	1.09 ± 0.07	209 ± 25	0.0053 ± 0.0004
K237R	58.7 ± 0.4	60.5 ± 0.2	59.00 ± 0.02	0.94 ± 0.04	67.3 ± 8.8	0.0143 ± 0.0017

a Error is based on the standard deviation for *T*_m_ values and on the standard error of the mean (SEM) for Michaelis-Menten parameters of biological replicates with *N* = 3.

Based on this biochemical characterization, we hypothesize that the improved performances of the H191E and K237R variants arise primarily from an increase in SAM affinity, which provides stabilization to the enzyme and the active sites. These mutations do not inherently increase the catalytic rate of the enzyme but rather enable a more efficient binding of SAM within the active site. This is particularly important for fluorinases because it is known that SAM binding is required for the full desolvation of the bound fluoride ions, which is the most energetically expensive part of the reaction.^33^ An increased affinity for SAM can help to address this constraint. While this provides a plausible explanation for the improved performance of H191E and K237R, it also raises the broader question of whether zero-shot models preferentially identify mutations that improve properties such as stability or substrate binding, rather than mutations that directly enhance catalytic chemistry. Our engineering campaign alone cannot determine whether such a tendency exists generally, and resolving this question will require systematic benchmarking. This uncertainty highlights the value of PRIZM’s model selection step, which empirically identifies the zero-shot model most informative for the targeted property.

H191 is located in the linker region between the two domains of the FlA monomer (Figure S12). Substitutions with glutamate could perturb the microenvironment, although the increased relative activity observed for H191K (Figure 3B) suggests that the effect is not dependent on the charge sign. Instead, introducing a charge at this position may facilitate better solvation of the linker, reduce hydrophobic frustration in this region, and stabilize the fold. This could ultimately lead to more stable active sites, which can better bind to SAM.

K237 is instead situated in the hexameric interface across from E247 (Figure S18). Here, the arginine substitution likely enhances the interactions with E247, either by strengthening existing hydrogen bonds or forming additional electrostatic contacts, thereby stabilizing the hexameric state of FlA. This is in agreement with our previous work that showed a trimeric FlA suffering a 200-fold increase in SAM *K*_m_ compared to the hexameric WT.^17^ Together with this work, these results highlight the catalytic relevance of this unique structural feature of fluorinases. However, to fully elucidate these mechanisms, future work should investigate how the changes to the electrostatics due to the K237R mutation affect the hexameric interface.

Finally, we were unable to elucidate the mechanisms of increased relative activity for H32L, G37D, and S166A. The last mutation was previously suggested to improve fluoride entry through the N-terminal tunnel,^14^ which we did not investigate here. For H32L and G37D, the effect may instead relate to improved resilience toward product inhibition, an influence highlighted by Slanska et al.,^15^ though such a characterization is outside of the scope of this study.

## Conclusion

In this study, we identified several FlA variants with enhanced performance using PRIZM, a novel zero-shot prediction-based approach for protein engineering. Using only PRIZM predictions, we achieved a 27.5% hit rate of single mutants with relative activity above WT. A combinatorial screen of the best variants resulted in a hit rate of 72.7%, while a targeted approach exhibited a 28.5% hit rate. Importantly, PRIZM is simple and versatile as it only needs a low-N data set and does not require any supervised learning. Moreover, despite the original low-N data set only having mutations in the hexameric interface, we were able to identify variants with increased activity across the entire protein sequence.

Our best PRIZM variant, H191E, and our best targeted variant, K237R, demonstrated 52.6 and 716.2% increases in relative activity at 62 °C, respectively. Our biochemical characterization indicated that this is a result of stabilization of the enzyme and enhanced SAM binding; Given that the highest increase in *T*_m,app_, ∼2.4 °C, was observed in the presence of SAM, we hypothesized that the mutations are not only stabilizing, but also improve binding to SAM, which then also contributes to the overall enzyme stability. This hypothesis was confirmed via kinetic analysis at elevated temperatures, which revealed a ∼3.6-fold decrease in SAM *K*_m_ and a ∼218% increase in catalytic efficiency for K237R. For H191E, we hypothesize that introducing a charge in the linker region could improve solvation and reduce hydrophobic frustration. Future work should investigate the mutational effects of both K237R and H191E, especially focusing on their enzyme stabilization.

Our analysis confirms the potential of the hexameric interface for modulating FlA activity. In addition, this study identified new distal regions important for FlA performance that do not directly interfere with the active site, such as the linker region between the two domains of the monomer and the second shell of the active site. Future exploration of these regions may provide insight into the mechanisms underlying the distal effects on FlA activity, with potential value for engineering FlA to enhance industrial performance.

## Materials and Methods

### Fluorinase Data Setup

The FlA low-N data was preprocessed by omitting all variants showing inconclusive relative activity. The structural model was created using AlphaFold3 ^34^ while the MSA was obtained using the EVcouplings alignment module^35^ with a bitscore cutoff of 0.3, a 90% identity threshold for redundancy removal, a 70% position filter, a 50% sequence fragment filter, and downweighting sequences with more than 80% sequence similarity, resulting in 9519 effective sequences (*N*_eff_ = 6528 after downweighting). The resulting MSA had a minimum and maximum identity of 13.8 and 98.0%, respectively, and a median identity of 22.6% (Figure S19). The binarization threshold was chosen to be a relative activity of 80% due to no variant exhibiting higher-than-wildtype activity. The *in silico* library of FlA contained all single-point mutants for the entire protein sequence and was created using our PRIZM pipeline, v1.0.1.^29^ This version contains ESM-1b,^31^ ESM-1v,^21^ ESM-2,^28^ ProGen2,^36^ ProtGPT2,^37^ RITA,^38^ UniRep,^23^ EVE,^39^ eUniRep,^23^ TranceptEVE,^22^ Tranception,^40^ ESM-IF1,^30^ and ProteinMPNN.^41^

### Materials

5′-fluoro-5′-deoxyadenosine (FDA) and 5′-chloro-5′-deoxyadenosine (ClDA) were kindly provided to us by Eray Bozkurt (Pablo Nikel, DTU Biosustain) and stored at −20 °C. All other materials used in this work are commercially available.

### Library Construction

The DNA library of hexameric interface mutants of the FlA gene from *Streptomyces* sp. strain MA37 (FlA^MA37^) was ordered from Neochromosome. The library was generated using saturation mutagenesis at the following seven hexameric interface residues: K237, D241, E247, R277, P285, Y286, and N287. The library was amplified via PCR and cloned into the pET28a­(+) backbone for protein expression using USER cloning.^42^ The USER reaction mixtures were transformed into *E. coli* and the mutants were purified as described in the following sections.

### Cloning and Transformation

The WT FlA gene from *Streptomyces* sp. strain MA37 (FlA^MA37^) was previously cloned into the pET28a­(+) backbone with an N-terminal His-tag and tobacco etch virus (TEV) cleavage site.^17^ Primers were designed using NEBaseChanger^43^ and introduced into the FlA gene with site directed mutagenesis via PCR, followed by incubation with the Kinase, Ligase, and DpnI (KLD) Enzyme Mix (New England Biolabs) for 1 h at room temperature (RT). The KLD reaction mixtures were then transformed into chemically competent DH5α *E. coli* cells (New England Biolabs) via heat shocking for 30 s at 42 °C, recovered in Super Optimal broth with Catabolite repression (SOC) medium for 1 h and then plated on agar plates containing kanamycin (50 μg/mL) overnight. Individual colonies were picked and grown in liquid cultures, miniprepped (Macherey-Nagel), and the resulting plasmids were sequenced via Sanger sequencing with T7 promoter and terminator primers to confirm the introduction of the desired mutation (Eurofins).

### Protein Expression and Purification

Plasmids with confirmed sequences were transformed via heat shock into BL21­(DE3) (Invitrogen) chemically competent *E. coli* as stated above. Expression cultures were inoculated 1:1000 with the overnight precultures and grown at 37 °C to an optimal density at 600 nm (OD_600_) of ∼0.8. Cultures were induced with 250 μM isopropyl β-d-1-thiogalactopyranoside (IPTG) and incubated overnight (at least 20 h) at 18 °C. Cells were harvested by centrifugation and either directly used in purification or flash frozen in liquid nitrogen and stored at −20 °C until purification.

All further steps during purification were performed either on ice or at 4 °C in a cold room. Pellets were resuspended in 15 mL Lysis Buffer (20 mM imidazole, 300 mM NaCl, 10 mM 4-(2-Hydroxyethyl)­piperazine-1-ethane-sulfonic acid (HEPES), 0.05% (v/v) Triton X-100, 10 μg/mL DNase, pH 7.4) and lysed via sonication for 3 min (15 s on/30 s off) with 60% amplitude with a 6 mm probe (Sonics & Materials Inc.). The lysate was then centrifuged and filtered.

The samples were purified using an ÄKTA Pure M fast protein liquid chromatography (FPLC) system (Cytiva Life Sciences) with a 1 mL HisTrap FF column (Cytiva Life Sciences). The samples were loaded on the column, washed with 20 mL 90% Buffer A (20 mM imidazole, 300 mM NaCl, 10 mM HEPES, pH 7.4) and 10% Buffer B (500 mM imidazole, 300 mM NaCl, 10 mM HEPES, pH 7.4), and eluted with 15 mL 40% Buffer A and 60% Buffer B into 1 mL fractions. Protein molecular weight (MW) and purity was characterized using SDS-PAGE on a NuPAGE 4–12% Bis-Tris polyacrylamide gel (Invitrogen). Peak fractions were pooled and buffer exchanged into Buffer C (150 mM NaCl, 50 mM HEPES, pH 7.4) using PD-10 desalting columns (Cytiva Life Sciences). Purified proteins were concentrated to a final concentration of 5 mg/mL using 30 kDa Amicon Ultra Centrifugal Filters (Merck Millipore), flash frozen in liquid nitrogen, and stored at −70 °C for further use.

### Enzyme Activity Assays

Protein aliquots were thawed on ice and the concentrations were measured via NanoDrop (Thermo Scientific) before diluting all samples to 2 mg/mL. A separate master mix was made for each replicate, with a final substrate concentration of 300 μM SAM and 75 mM KF in Reaction Buffer (150 mM NaCl, 10 mM HEPES, pH 7.8). The master mixes were aliquoted in PCR tube strips, and the protein variants were added simultaneously with a multichannel pipet to a final hexamer concentration of 1 μM, based on the hexameric molecular weight (204 kDa). The reactions were incubated at 37 °C (unless otherwise stated) and quenched via heat inactivation at 95 °C for 5 min. For the relative activity assays, the 3 h time point, the protein was added to the master mix on ice before being transferred to a thermocycler, while the protein was added in the preheated samples for the 2 min time point. The samples were diluted 2× and filtered using 0.45 μm 96-well plate Durapore membranes (Millipore) and run on the HPLC. For quantification of SAM conversion, the FDA concentration was divided by the total amount of FDA and SAM (FDA concentration and SAM degradation product concentration).

For the product inhibition assays, the reactions were performed as stated above, except for the addition of exogenous FDA at the start of the reaction. Protein concentrations were measured using the Bicinchoninic Acid (BCA) kit (Novagen). Quantification was performed via HPLC with standard curves for quantification of molar amounts of FDA and SAM. FDA concentrations were corrected to account for the initial amount of FDA added by subtracting the area of the FDA peak in a no enzyme control.

For determining the Michaelis–Menten parameters of the variants, reactions were performed using 1 μM of enzyme, based on the hexameric molecular weight, 10–1200 μM SAM, and 75 mM KF in Reaction Buffer. The reactions were performed at 57 °C for 15s and 30s and quenched via heat inactivation at 95 °C for 5 min. Three replicates were performed on separate days. Michaelis–Menten kinetics were assumed to determine *k*_cat_ and *K*_M_. The velocity of the enzyme was determined for each SAM concentration using linear regression and the Michaelis–Menten equation was then fit to the velocity-concentration data set using GraphPad Prism (version 10.6.1). Parameters were obtained by fitting each replicate independently, and reported values are mean ± SEM.

### High Performance Liquid Chromatography (HPLC)

The reaction compositions were analyzed by Reversed-Phase HPLC using an Ultimate 3000 Series HPLC (Thermo Fisher Scientific) and a ZORBAX RR Eclipse Plus C18 Column (100 mm × 4.6 mm, 3.5 μm) (Agilent) maintained at 30 °C. Milli-Q water +0.1% (v/v) formic acid and acetonitrile were used as mobile phases A and B, respectively, with the following method in percentages of mobile phase B at 1 mL/min: 0–1.5 min 5–12% (gradient), 1.5–2.5 min 12%, 2.5–4.5 min 12–30% (gradient), 4.5–5 min 30–70% (gradient), 5–5.5 min 70–5% (gradient), 5.5–7 min 5%. Chromatograms were recorded at 260 nm and integrated using the Chromeleon software (version 7.2.9). Using this method, FDA elutes at 2.64 min, a SAM degradation product elutes at 3.81 min, and vanillin elutes at 6.35 min (Figure S20). Standard curves correlating FDA and SAM concentrations with the ratios of their absorbances at 260 nm were generated using solutions of FDA or SAM from 0.5 to 30 μM and 0.5–300 μM, respectively (Figure S21). SAM cannot be measured directly on the HPLC due to its instability, so the standard curve was created using the degradation product mentioned above.

### Differential Scanning Fluorimetry (DSF)

The melting temperatures of the purified enzymes were determined using the Protein Thermal Shift Dye Kit (Thermo Fisher Scientific) and a qPCR QuantStudio 5 Real-Time PCR machine (Applied Biosystems). Immediately before the experiment, 50 μL of 2× dye was mixed with 50 μL of enzyme at 1 mg/mL. The melting temperatures were measured in the following conditions: (1) in Reaction Buffer, (2) in Reaction Buffer with 75 mM KF, and (3) in Reaction Buffer with 300 μM SAM. The samples were incubated at 25 °C for 2 min, then the temperature was linearly increased to 99 °C. All data analysis was performed using Protein Thermal Shift Software v1.4.

## Supplementary Material





## Data Availability

All data, analysis scripts, and code related to PRIZM used in this study are openly available at GitHub: https://github.com/daha-la/PRIZM. The version of the PRIZM workflow used in this study corresponds to the GitHub release v1.0.1, which contains the complete codebase, analysis scripts for reproducing the figures, and the data sets underlying the study.

## Notes

Experimental relative-activity characterization of initial FlA variants, PRIZM variants, combinatorial mutants, and targeted variants, together with zero-shot scores of all FlA single mutants (XLSX)
Correlation between zero-shot scores of FlA *in silico* library from best models; comparison of double and single mutant zero-shot scores; structural illustrations of FlA; relative activity measurements of single and combinatorial variants; comparison of WT and top FlA variants (Δ*T*_m,app_), temperature optimum, relative activity at different temperatures after 2 and 10 min; FDA inhibition analysis; representative Michaelis Menten curve, representative HPLC trace; distribution of sequence identities in initial MSA; SAM and FDA calibration curves; and supporting data sets including variant libraries and multiple sequence alignments (PDF)

## Abbreviations

ClDA: 5′-chloro-5′-deoxyadenosine
FDA: 5′-fluoro-5′-deoxyadenosine
FlA: fluorinase from *Streptomyces* sp. MA37
HPLC: high-performance liquid chromatography
ML: machine learning
PDB ID: Protein Data Bank Identifier
PRIZM: Protein Ranking using Informed Zero-shot Modeling
SAM: S-adenosyl-l-methionine
WT: wildtype
