# Supplementary material for: Data-Efficient Distal Engineering of Fluorinase Using Zero-Shot Models
Source: Chem Bio Eng. 2026 Aug 5;3(10):865–75. doi: 10.1021/cbe.6c00028 (PMC13697940; doi:10.1021/cbe.6c00028)
Supplement: Supplementary file 1 [file be6c00028_si_002.pdf]

Supporting Information

Data-efficient distal engineering of fluorinase using zero-shot models

David Harding-Larsen[1,‡], Brianna M. Lax[1,‡], Christopher Kurt Weingarten[1], Aboubakar Sako[1], Stanislav Mazurenko[2,3,], Ditte Hededam Welner[1,*]*

[1] The Novo Nordisk Foundation Center for Biosustainability, Technical University of Denmark, Søltofts Plads 220, 2200, Kongens Lyngby, Denmark

[2] Loschmidt Laboratories, Department of Experimental Biology and RECETOX, Faculty of Science, Masaryk University, Kamenice 5, 625 00, Brno, Czech Republic

[3] International Clinical Research Center, St. Anne's University Hospital, Pekařská 664/53, 602 00, Brno, Czech Republic

‡ Contributed equally to this work
* Corresponding authors, mazurenko@mail.muni.cz & diwel@dtu.dk

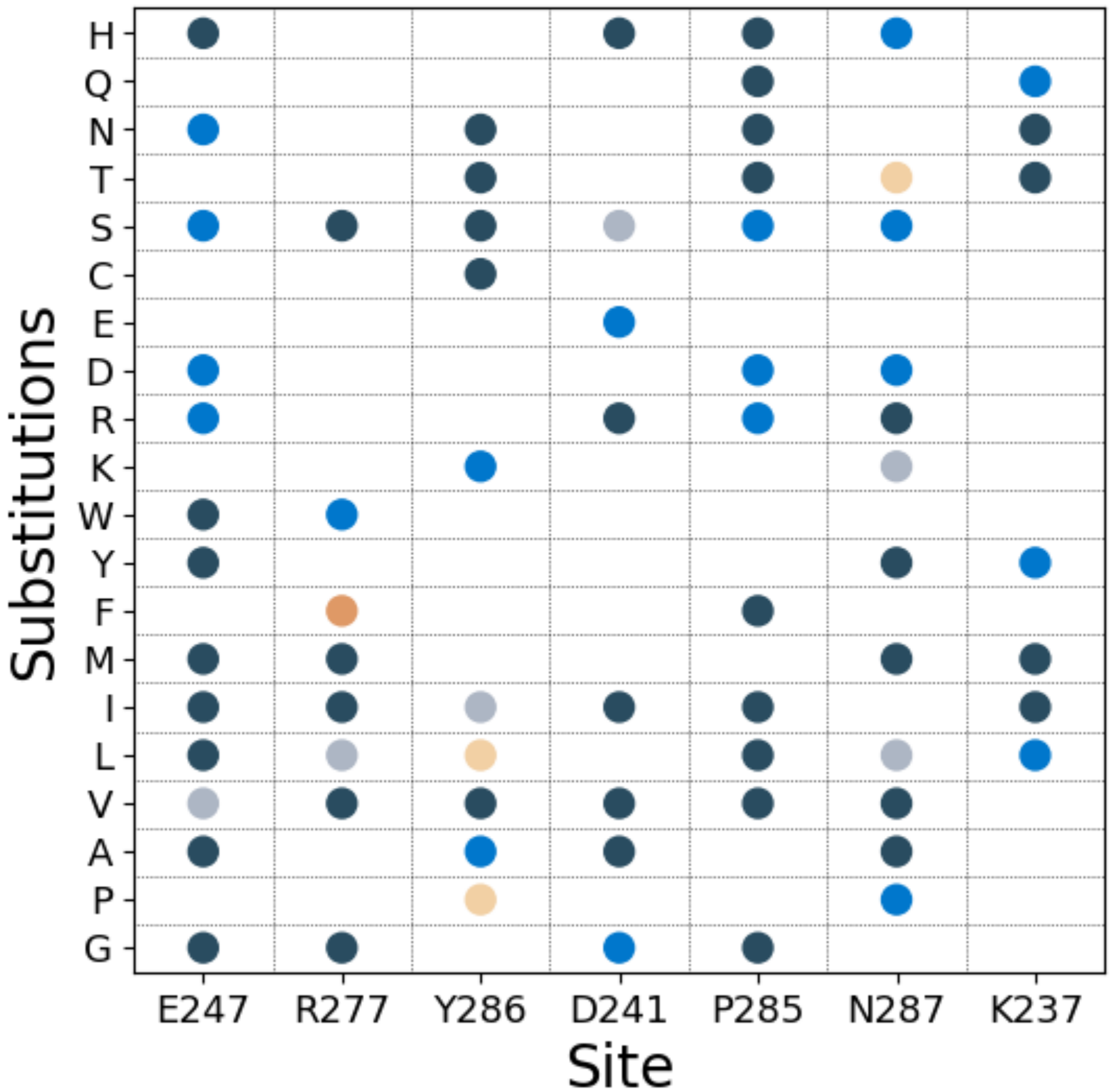

Figure S1. Distribution of mutations across the seven hexameric interface residues contained in the FlA low-N dataset (N = 40). Number of occurrences are denoted by color: 1 = dark blue, 2 = blue, 3 = grey, 4 = beige, 5 = orange.

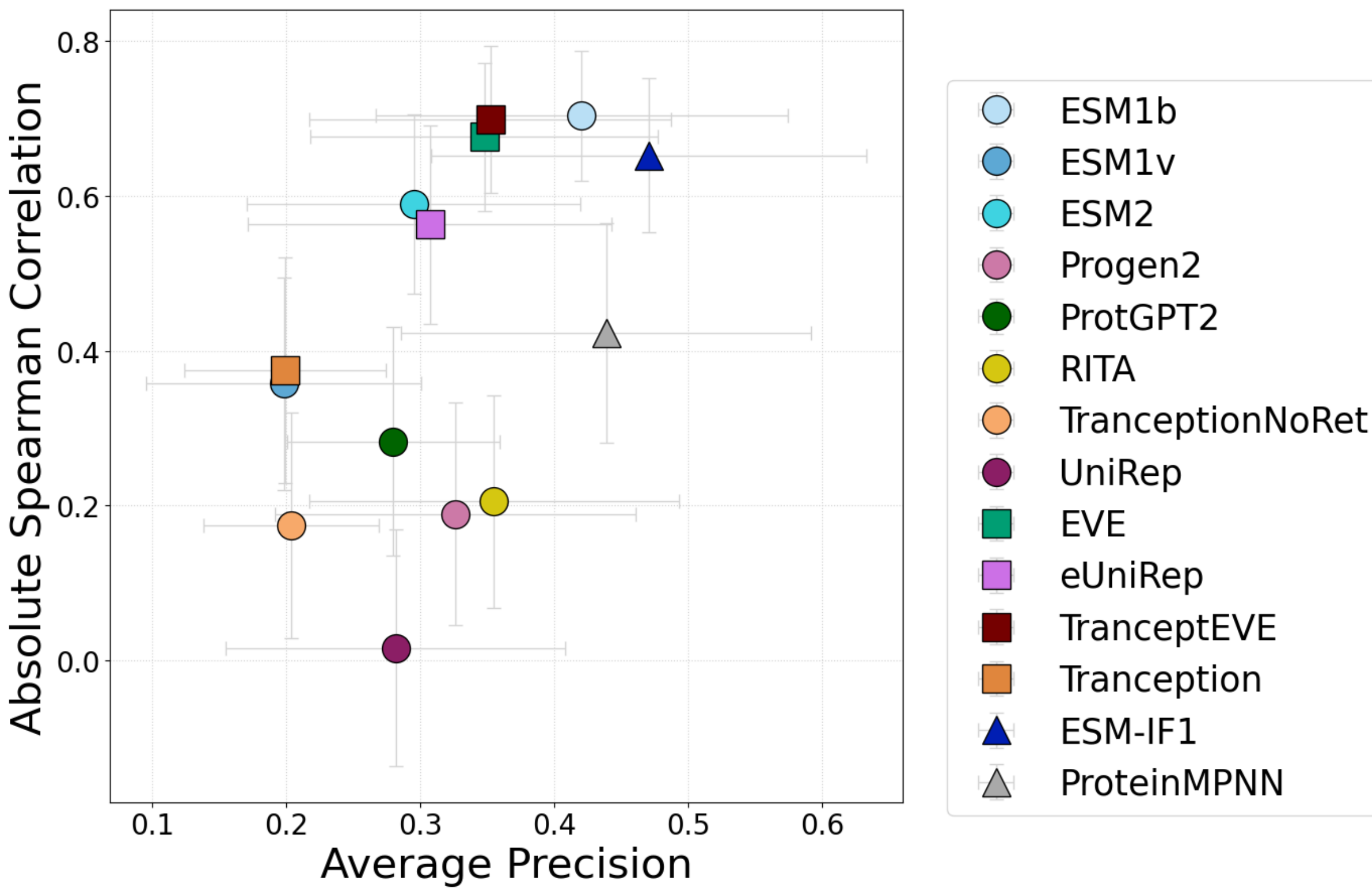

Figure S2. Spearman correlation and average precision for zero-shot scores and experimental values for the different zero-shot models of PRIZM (circle = sequence-based model, square = MSA-based model, and triangle = structure-based model). Error bars indicate the standard deviation estimated from bootstrapping the dataset (N = 1000).

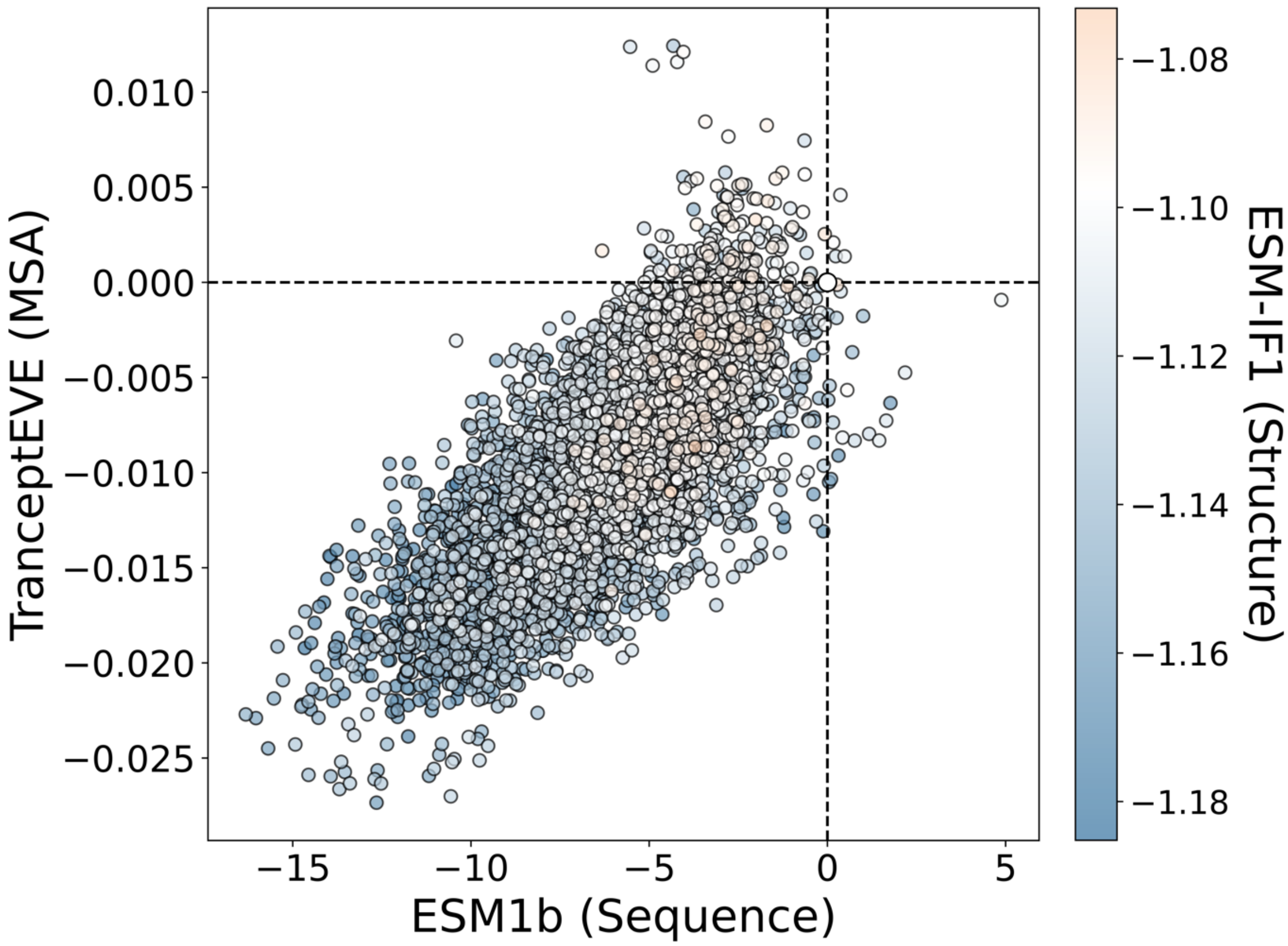

Figure S3. Zero-shot scores of all single-point variants using the best models for each category identified by PRIZM using the FlA low-N dataset. WT is denoted by dashed line and white color, while variants predicted by ESM-IF1 to be better or worse than WT are colored orange or blue, respectively.

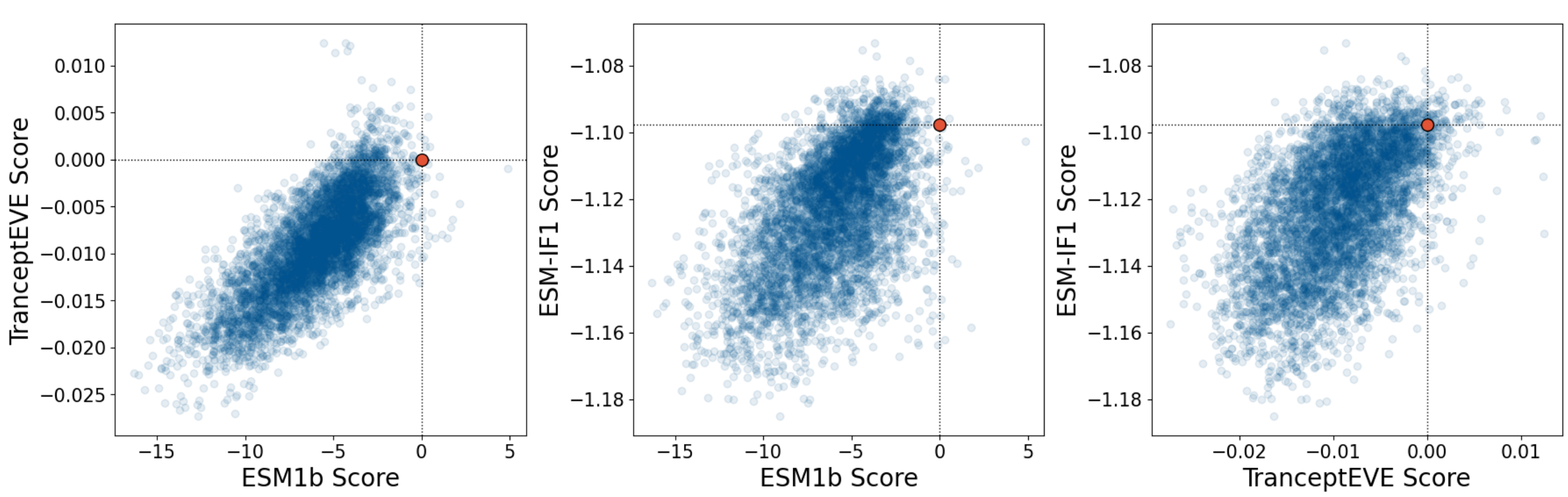

Figure S4. Pairwise comparison of zero-shot scores of all single-point variants using the best models for each category identified by PRIZM using the FlA low-N dataset. WT is denoted by dashed lines and red color.

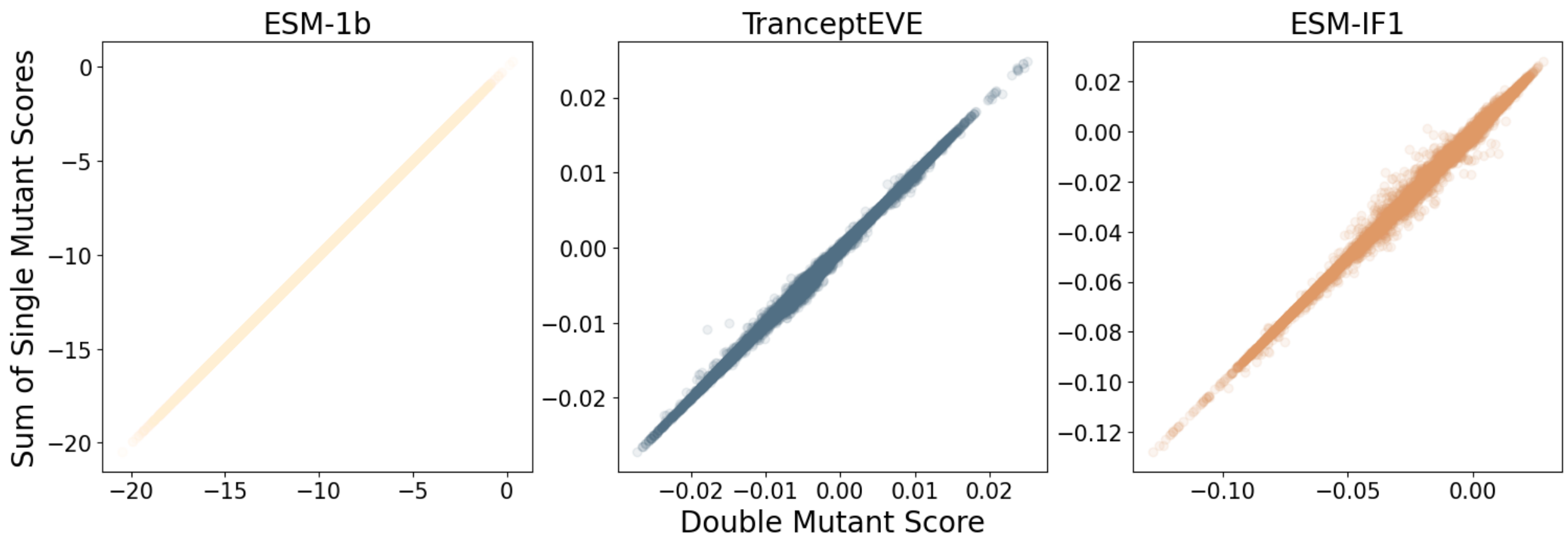

Figure S5. Comparison of double mutant scores vs. sum of single mutant scores for the three best models. Each double mutant score is matched to its corresponding combination of single mutants. The ESM-IF1 scores are calculated by subtracting WT score from both double mutant and each single mutant. Pearson correlation is 1.0 for ESM-1b, 0.9992 for TranceptEVE, and 0.9989 for ESM-IF1 (N = 32850).

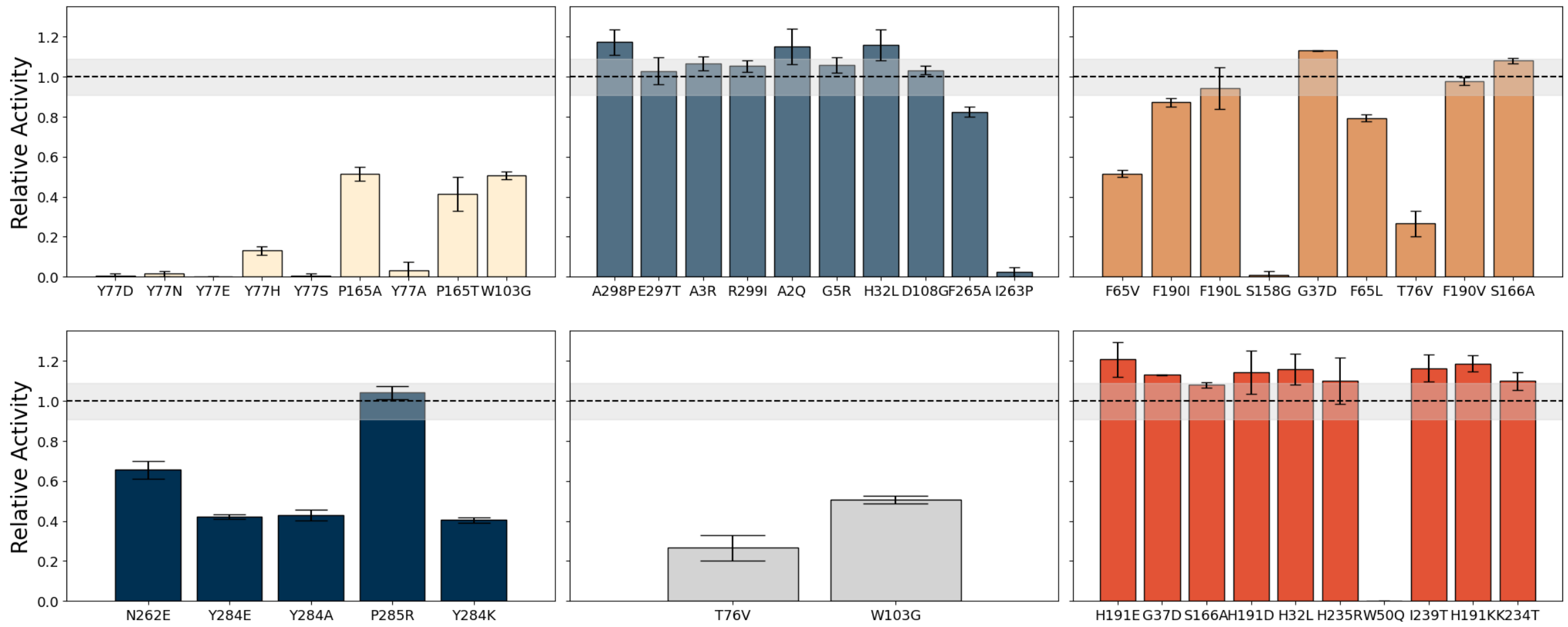

Figure S6. Relative activity after two minutes of single mutants selected using the PRIZM workflow. The top row shows variants selected using a greedy top 10 approach for ESM-1b (beige), TranceptEVE (blue), and ESM-IF1 (orange), while the bottom row shows the results of single mutants predicted to be better than WT by two models (grey = combination of ESM-1b and ESM-IF1, dark-blue = combination of ESM-1b and TranceptEVE, and red = combination of TranceptEVE and ESM-IF1). Dashed lines denote the WT value of 1. Error bars indicate standard deviation of biological replicates (N=3).

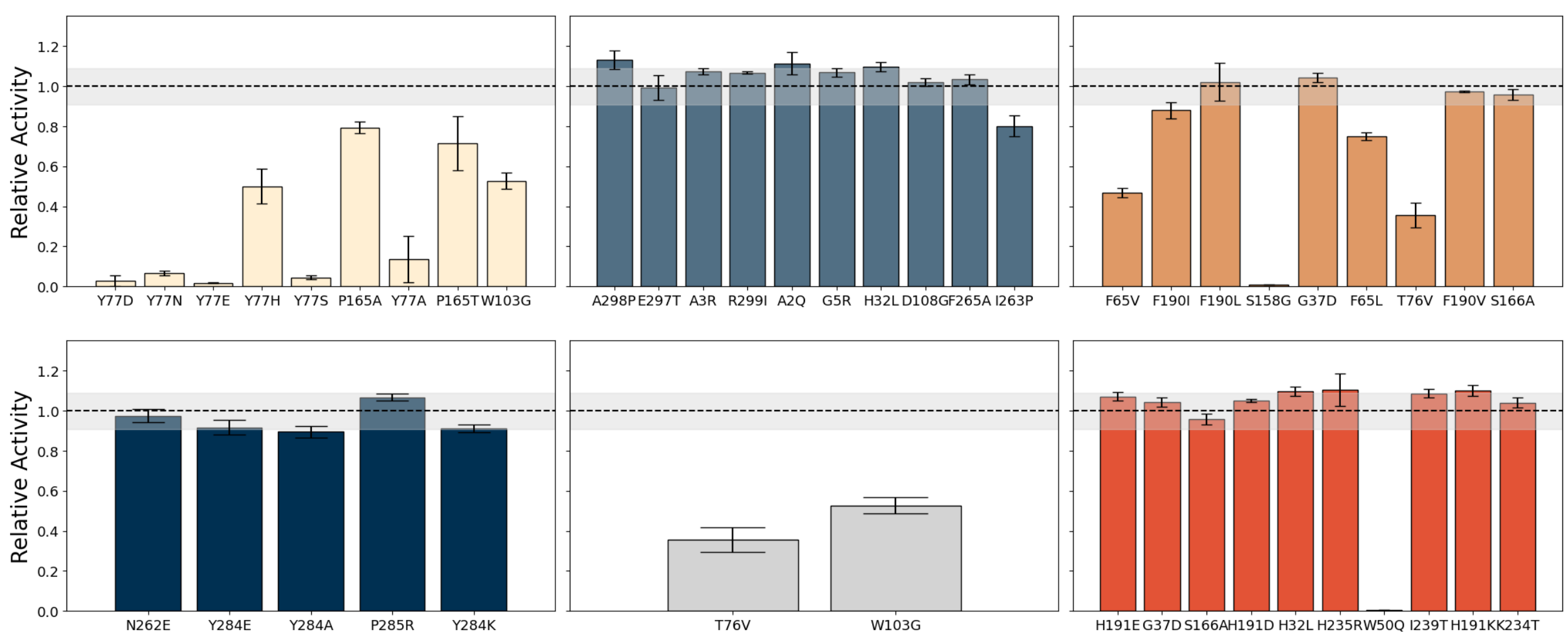

Figure S7. Relative activity after three hours of single mutants selected using the PRIZM workflow. The top row shows variants selected using a greedy top 10 approach for ESM-1b (beige), TranceptEVE (blue), and ESM-IF1 (orange), (grey = combination of ESM-1b and ESM-IF1, dark-blue = combination of ESM-1b and TranceptEVE, and red = combination of TranceptEVE and ESM-IF1). Dashed lines denote the WT value of 1. Error bars indicate standard deviation of biological replicates.

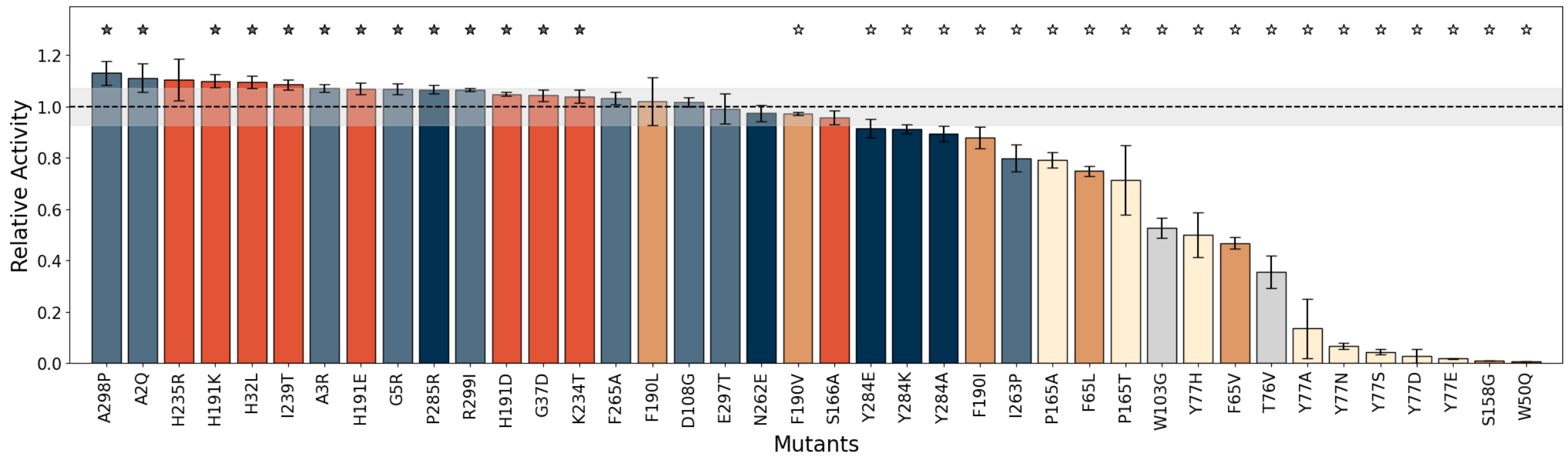

Figure S8. Relative activities after three hours of single mutants from different selection criteria: beige = ESM-1b, light-blue = TranceptEVE, orange = ESM-IF1, grey = combination of ESM-1b and ESM-IF1, dark-blue = combination of ESM-1b and TranceptEVE, and red = combination of TranceptEVE and ESM-IF1. Although certain variants were selected based on multiple selection criteria, each is displayed only once in this figure (for a comprehensive overview of all selections, refer to Figure S5). The dashed line denote the WT value of 1. Error bars indicate standard deviation of biological replicates. Stars denote variants with relative activity at two minutes significantly different from WT (one-sided Welch test with α = 0.05, grey = better than WT, white = worse than WT).

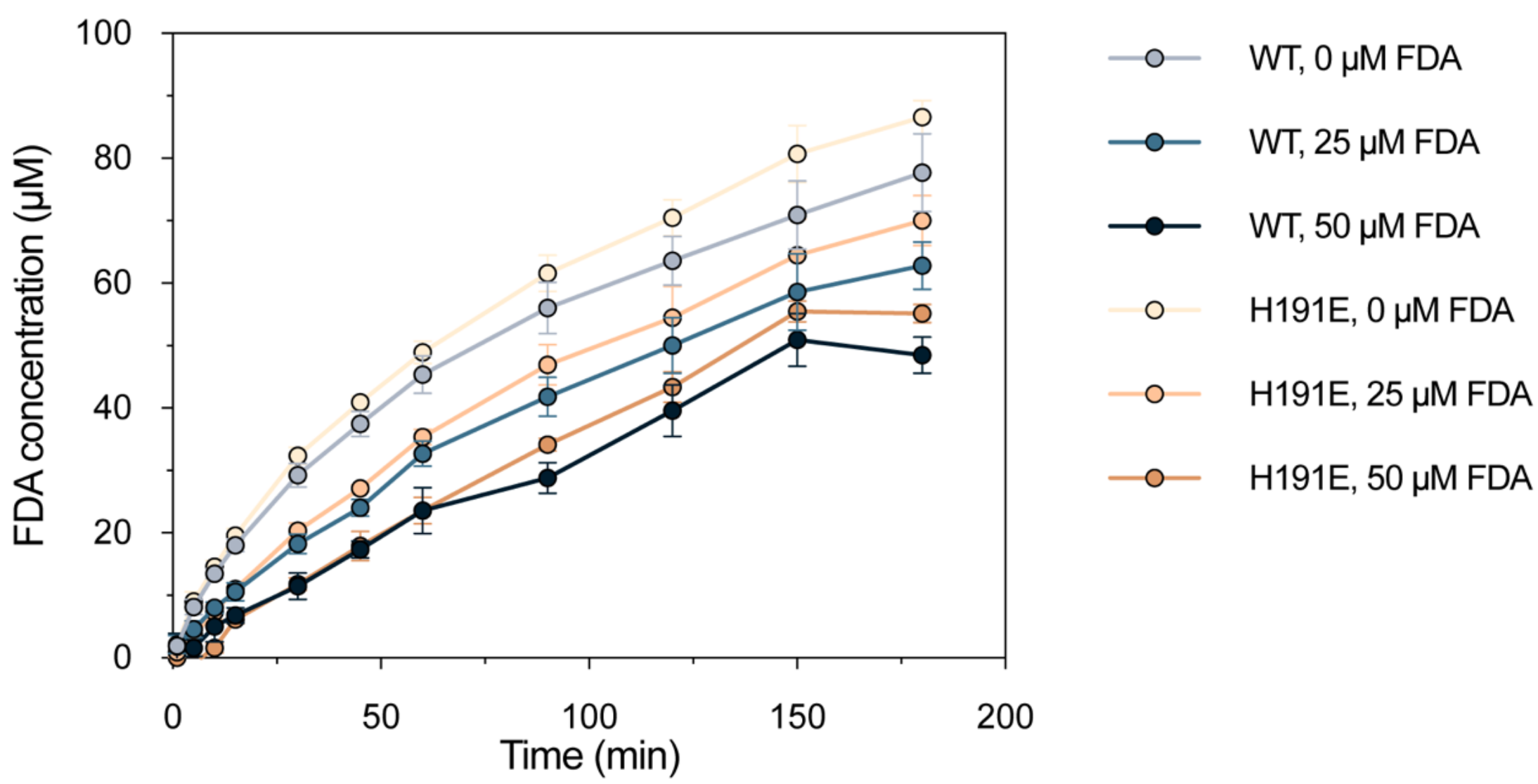

Figure S9. Enzymatic activity of FlA WT and H191E corresponding to analytical yields of FDA produced in the presence of exogenous FDA added at the beginning of the reaction, as denoted in the legend. The FDA concentration was corrected to account for the initial amount of FDA added by subtracting the area of the FDA peak in a no enzyme control. Error bars indicate the standard deviation of biological replicates with N = 3.

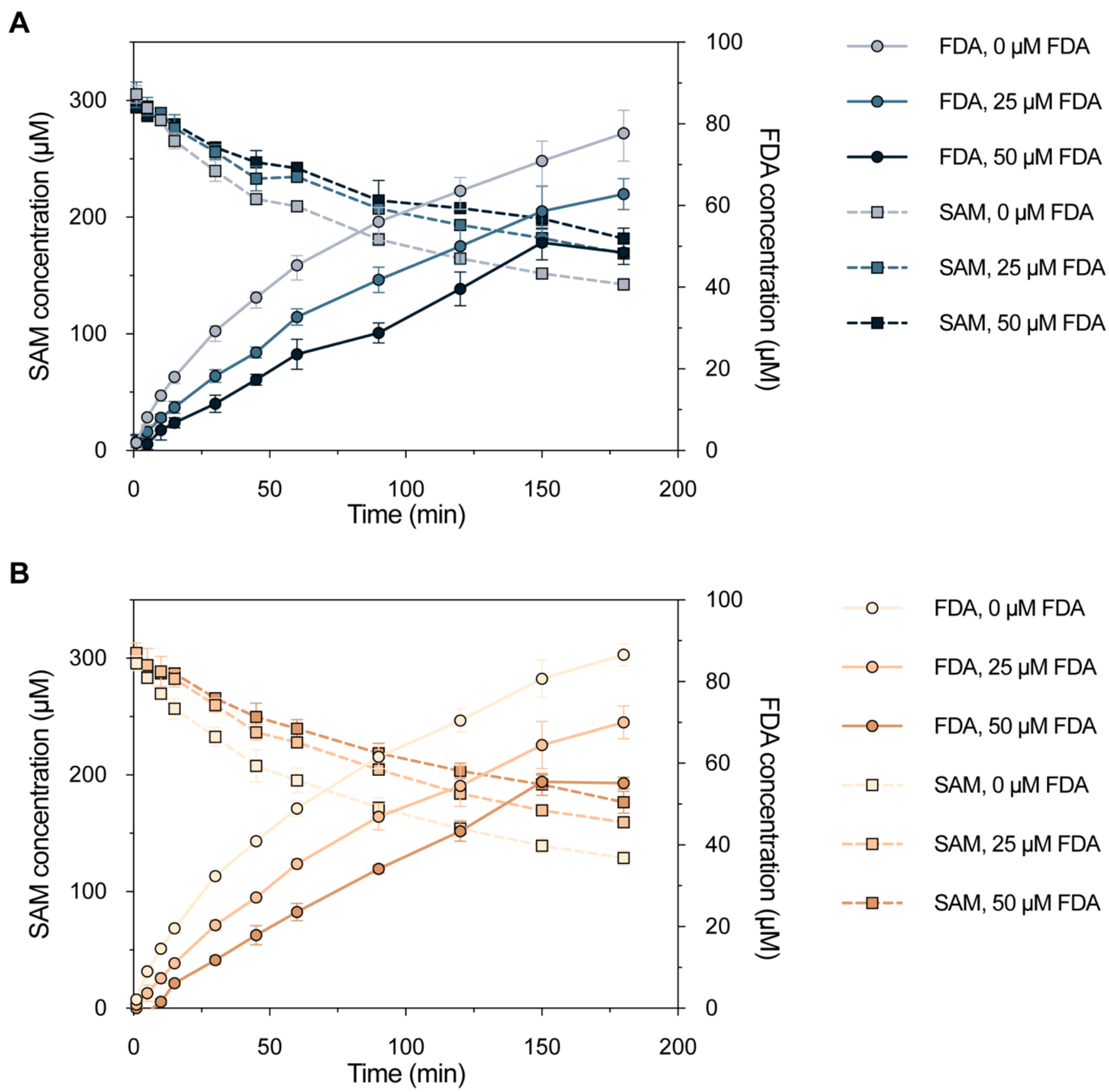

Figure S10. Analytical amounts of FDA produced (right y-axis) and SAM remaining (left y-axis) in the presence of exogenous FDA added at the beginning of the reaction, as denoted in the legend, for **A** FlA WT, and **B** mutant H191E. The FDA concentration was corrected to account for the initial amount of FDA added by subtracting the area of the FDA peak in a no enzyme control. Error bars indicate the standard deviation of biological replicates with N = 3.

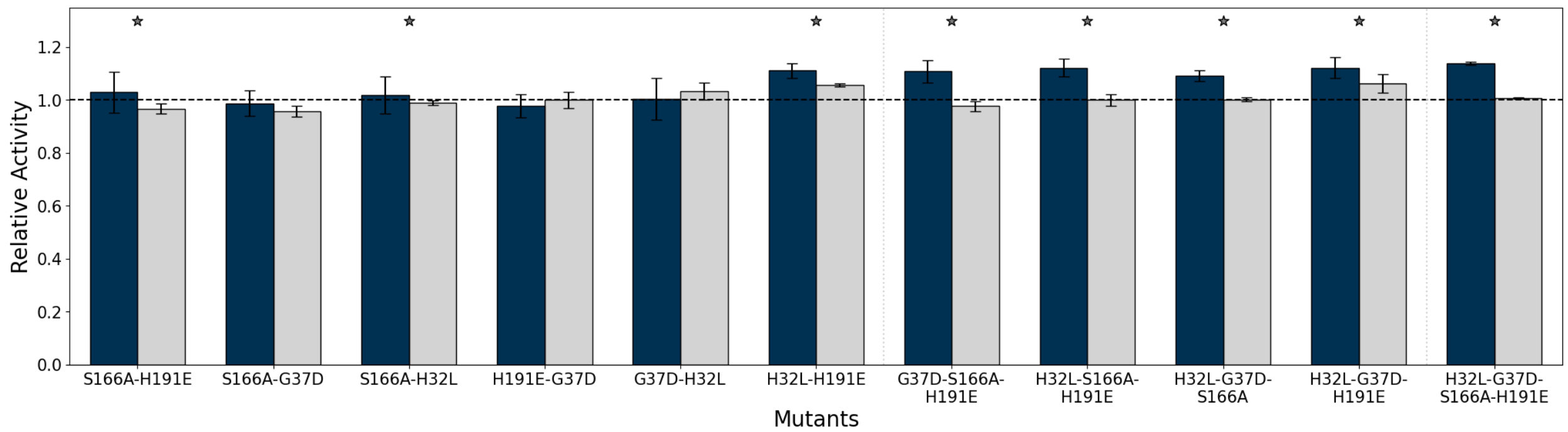

Figure S11. Relative activities of combinatorial mutants after two minutes (blue) and three hours (grey). The dashed line denotes the WT value of 1. Error bars indicate standard deviation of biological replicates. Variants with relative activity significantly above WT at two minutes (one-sided t-test with α = 0.05) are denoted by a star.

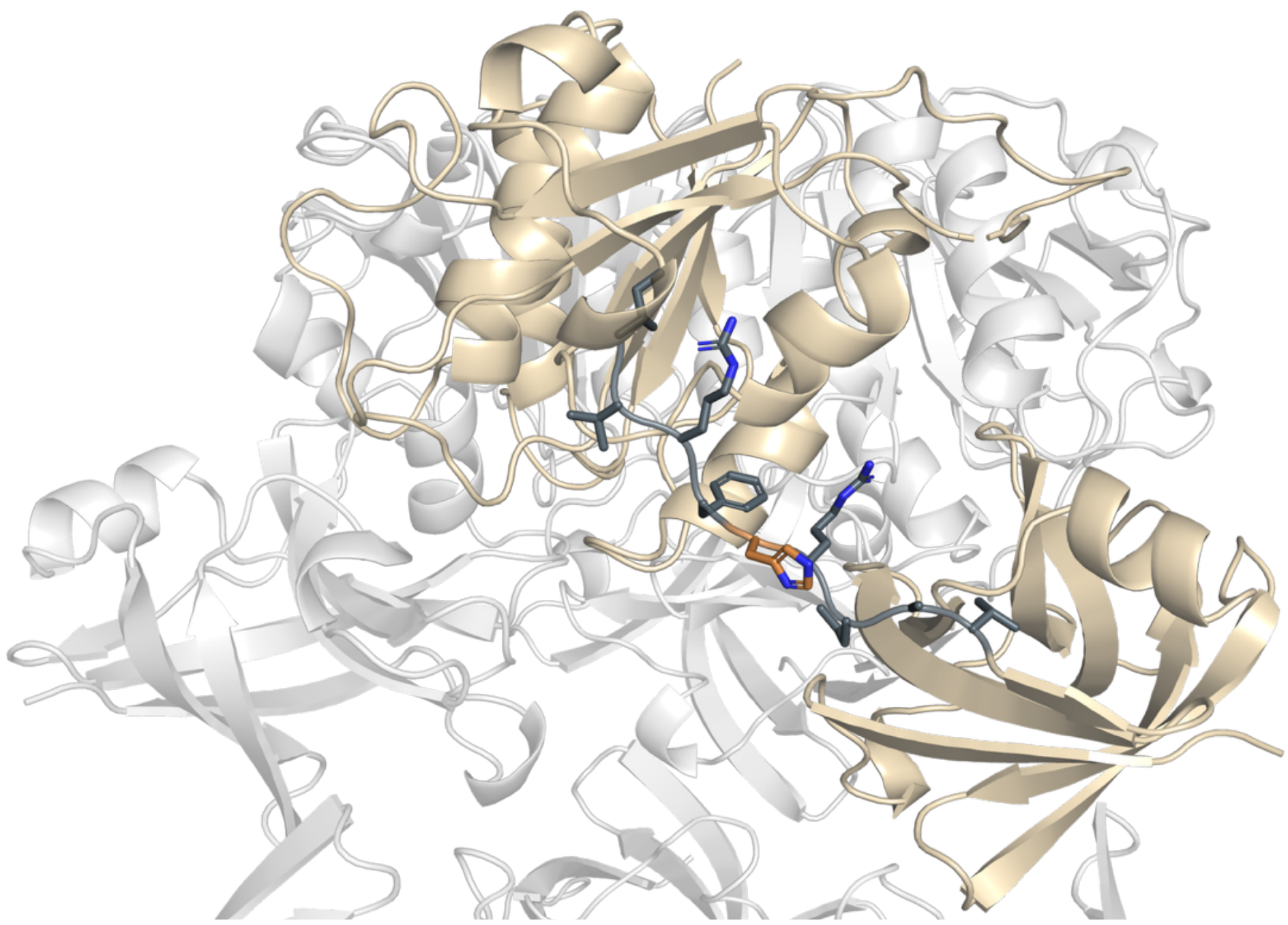

Figure S12. The FlA crystal structure (PDB ID: 5B6I) with the monomeric unit colored beige, while the linker region between monomer domains and the H191 residue are shown as sticks and colored grey and orange, respectively.

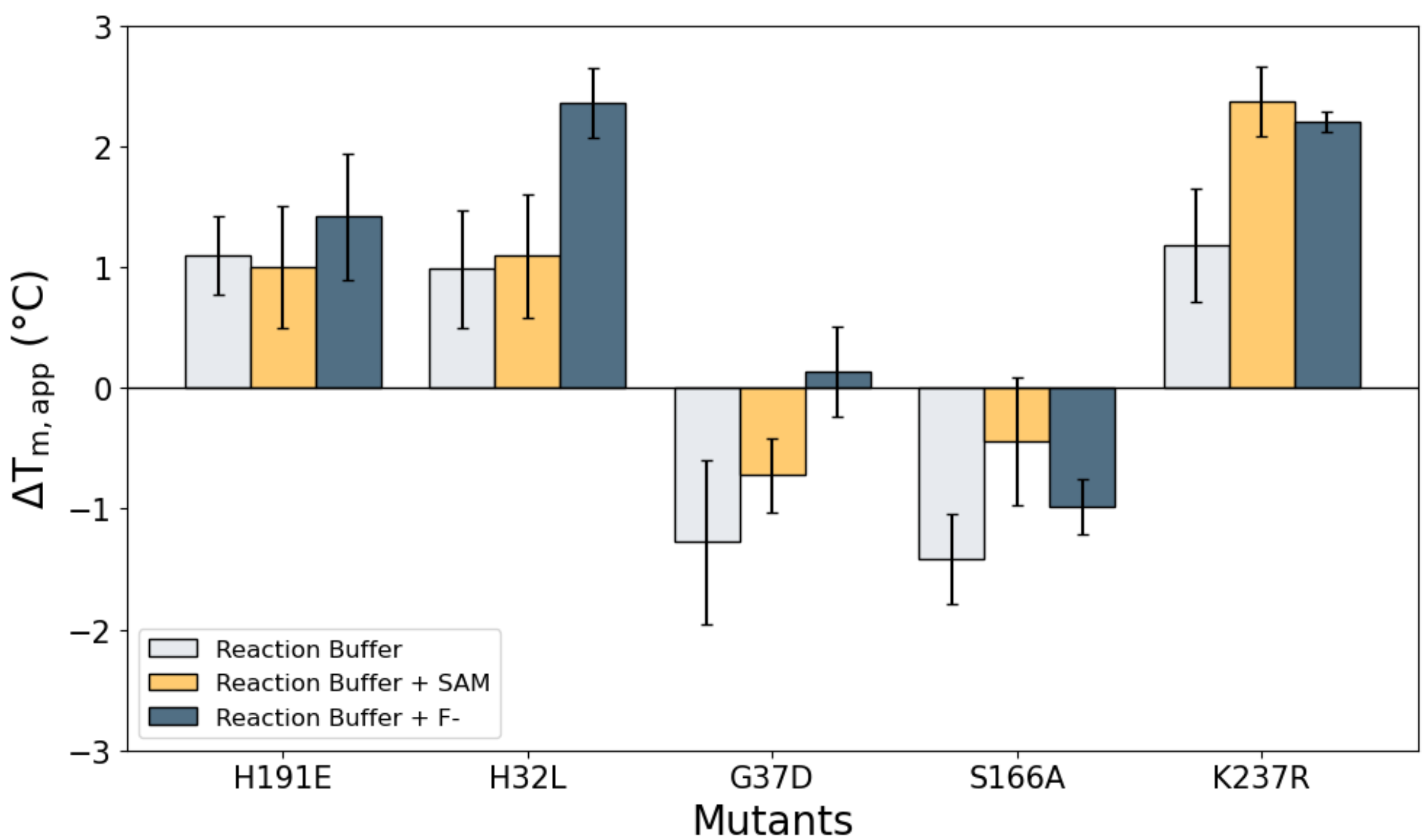

Figure S13. $\Delta T_{m,app}$ of WT and top FlA variants at different conditions: reaction buffer alone (grey), with SAM (yellow), or with F^- (blue). Error bars indicate the standard deviation of biological replicates with N = 3.

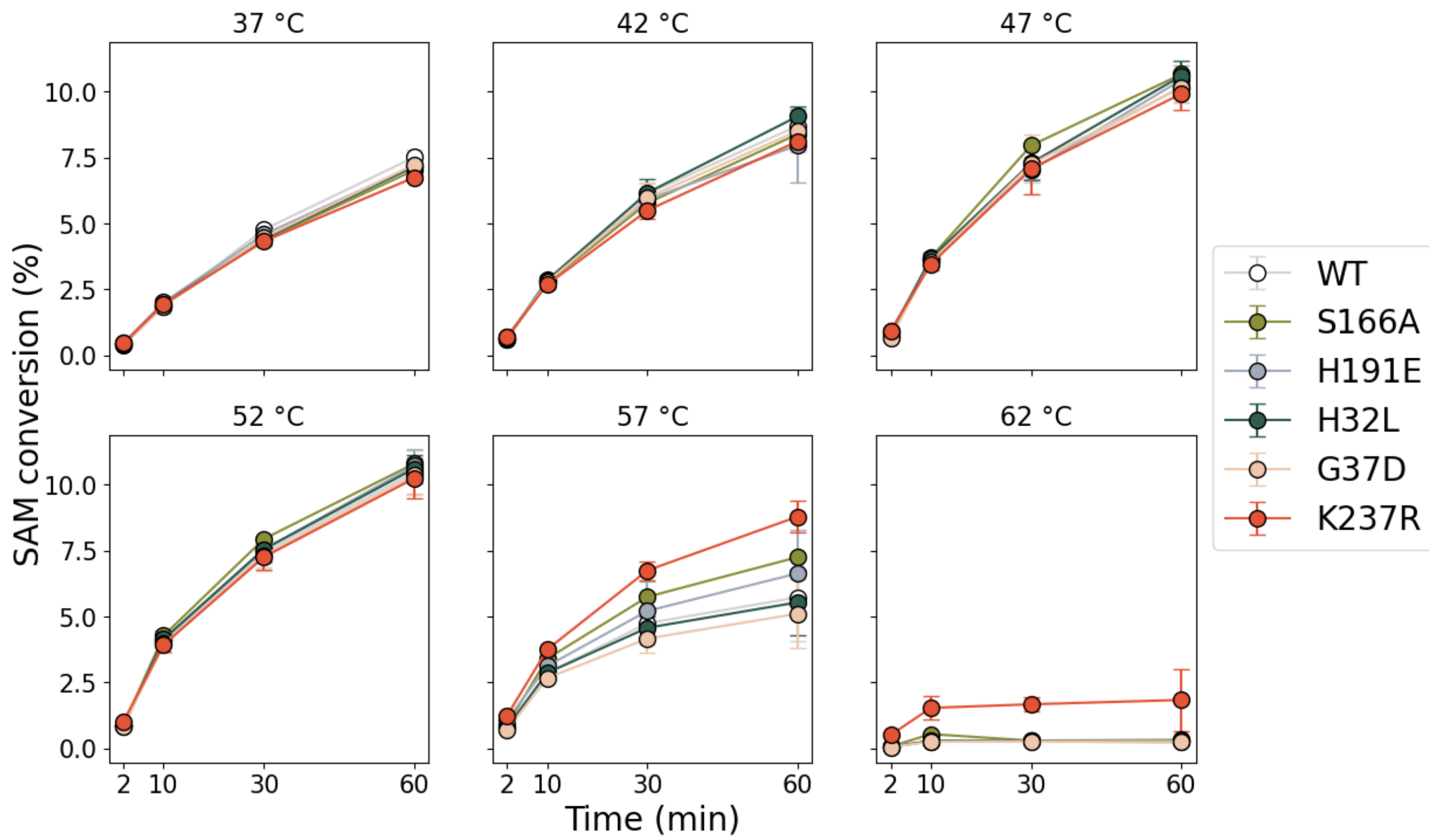

Figure S14. SAM conversion (%) for WT and top FlA variants at different temperatures and time points. Error bars indicate the standard deviation of biological replicates with N = 3.

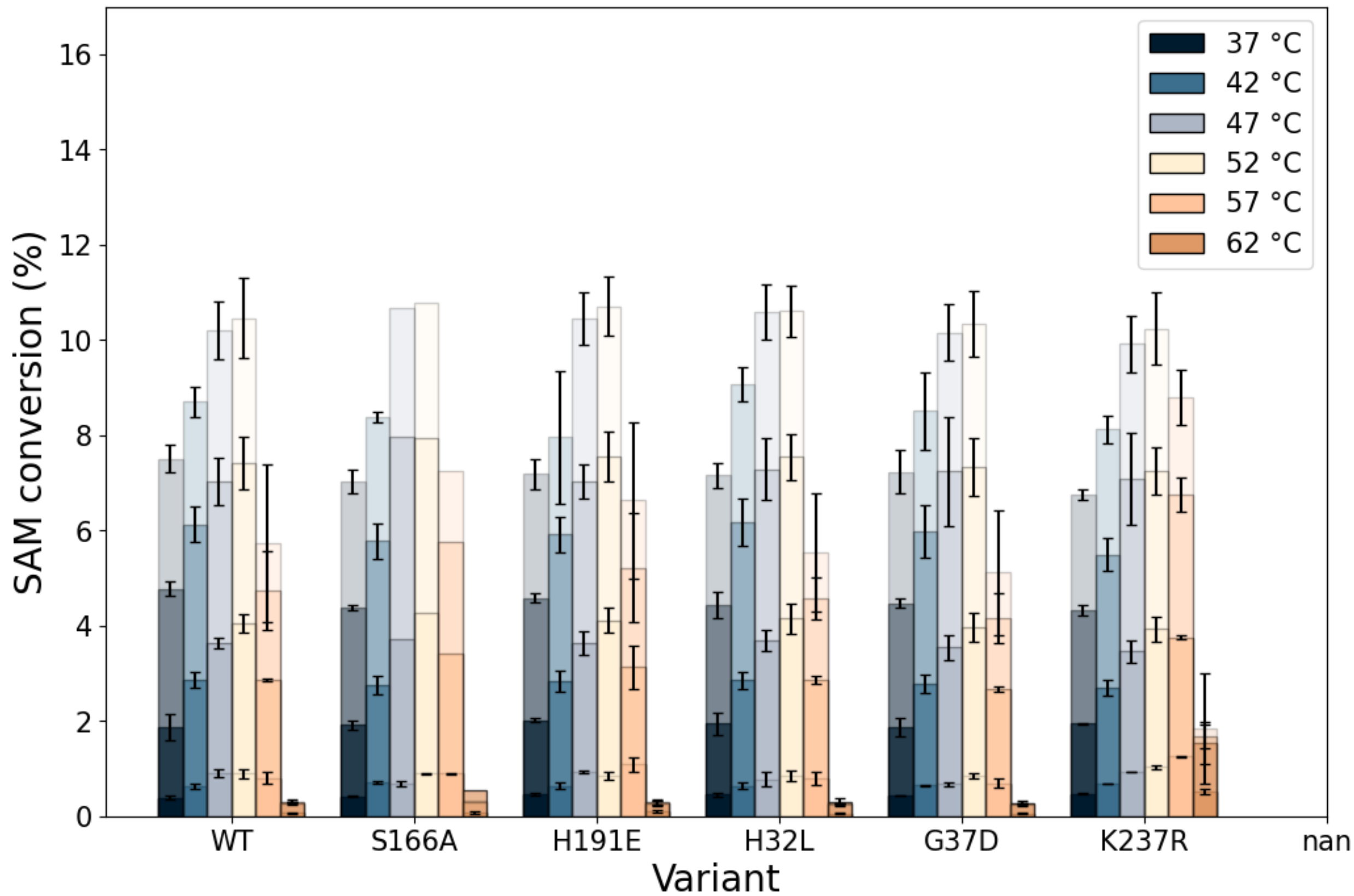

Figure S15. SAM conversion (%) for WT and top FlA variants at different temperatures and time points. Error bars indicate the standard deviation of biological replicates with N = 3.

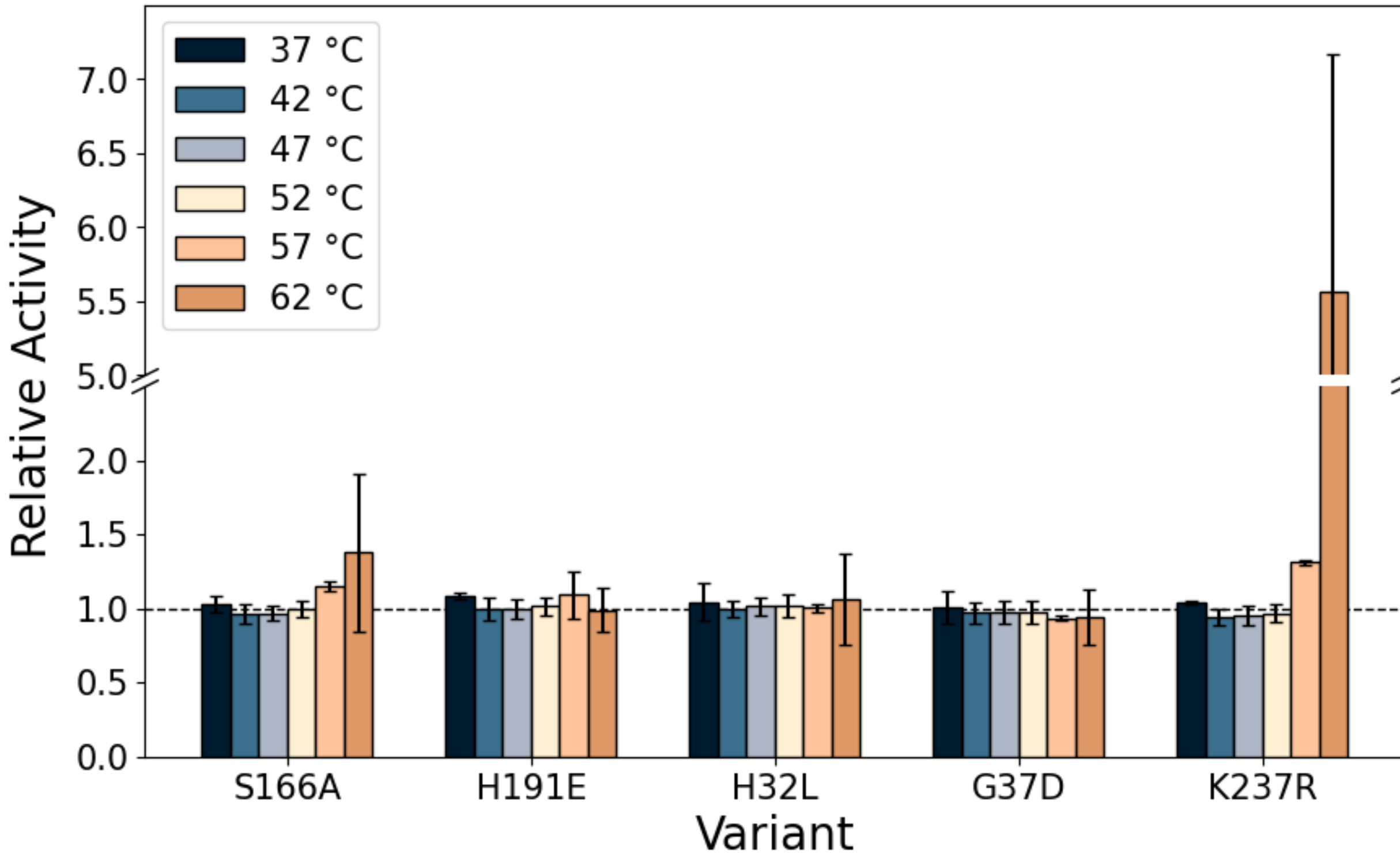

Figure S16. Relative activity of WT FlA and top FlA after ten minutes at different temperatures. Error bars indicate the standard deviation of biological replicates with N = 3, while the dashed line indicates the relative activity of 1.

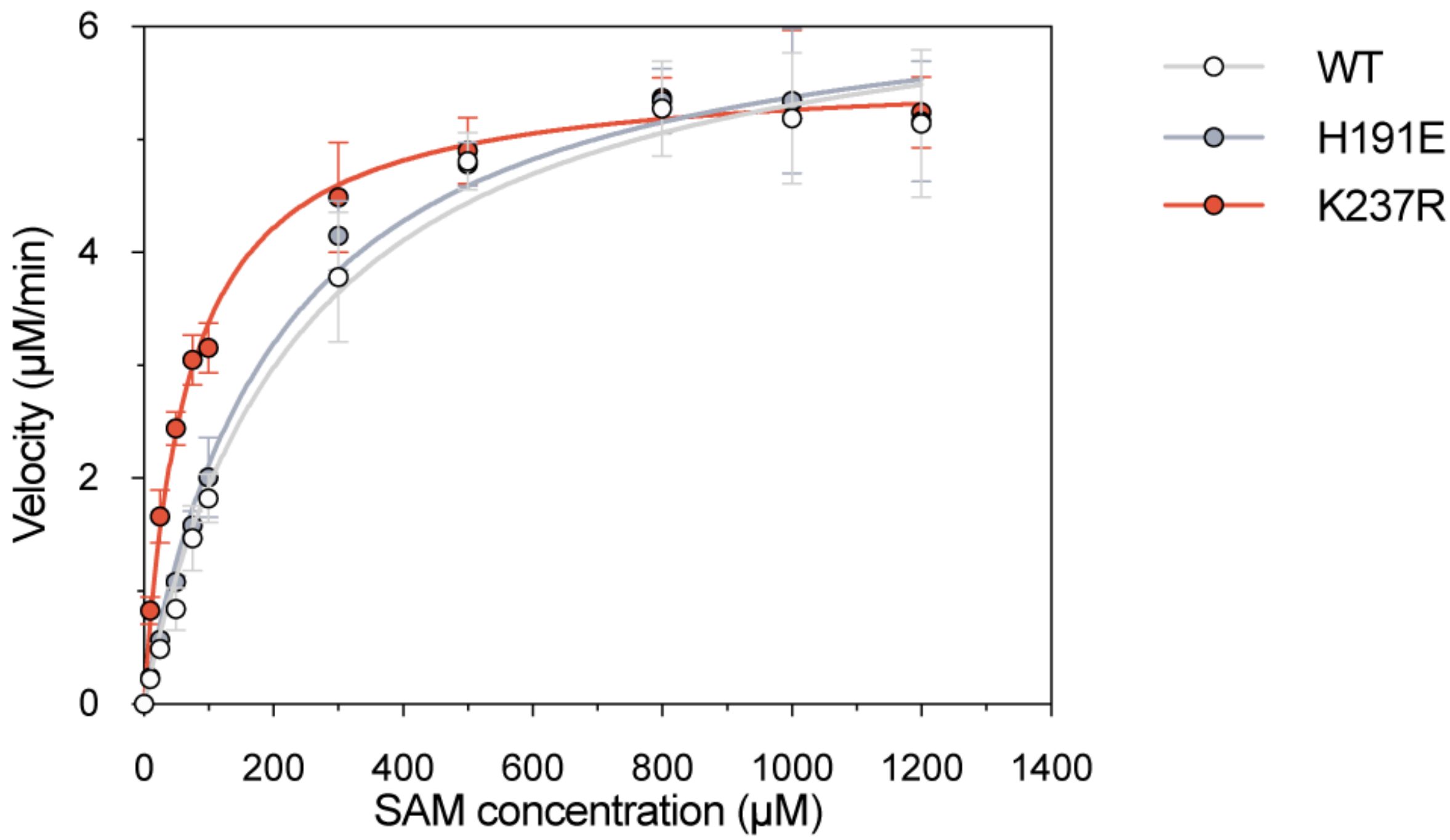

Figure S17. Michaelis-Menten kinetics of WT, H191E, and K237R measured across increasing SAM concentrations. Points show mean initial velocities, error bars indicate the standard deviation of biological replicates with N = 3, and curves are nonlinear Michaelis-Menten fits using GraphPad Prism version 10.6.1.

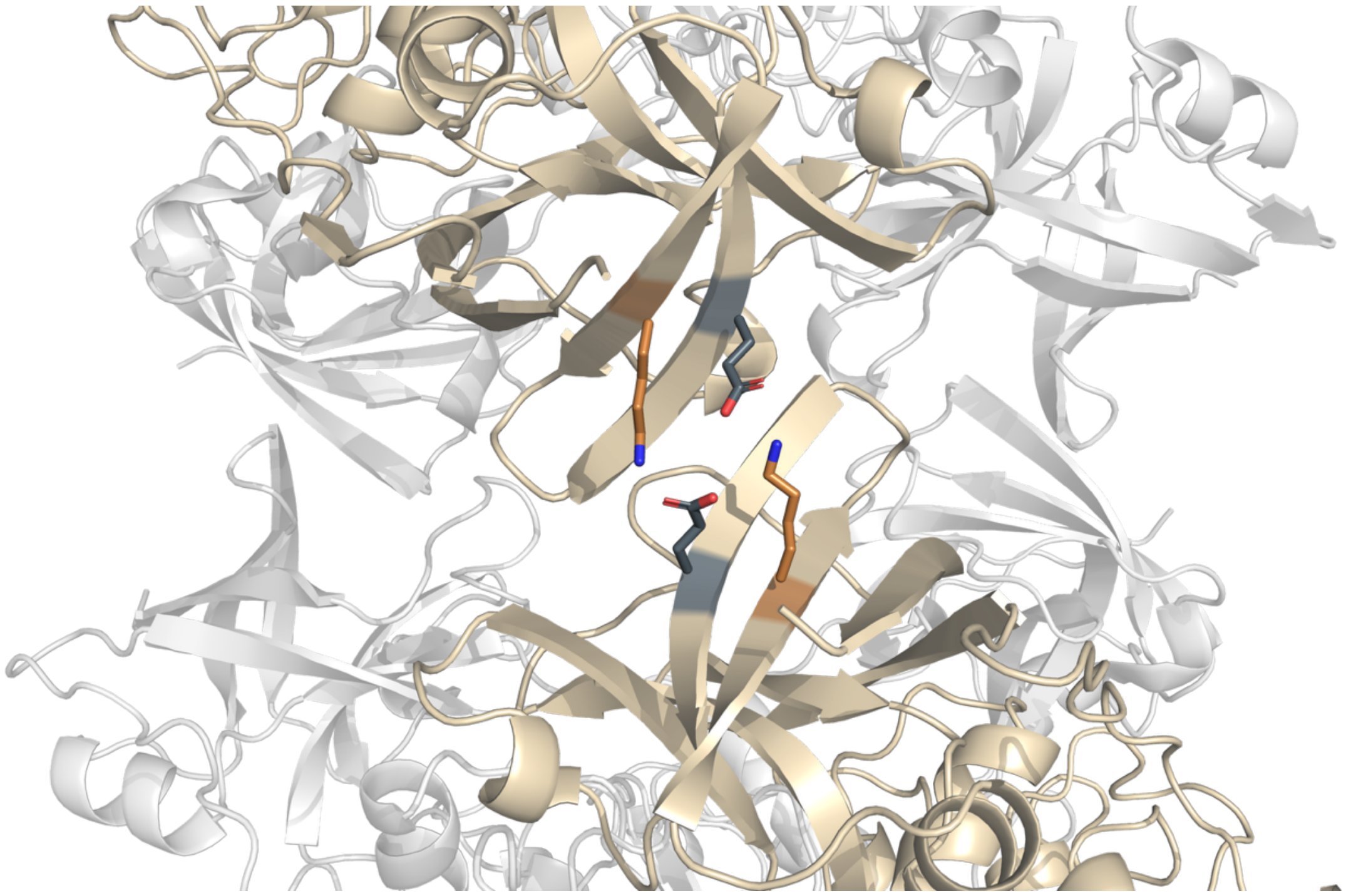

Figure S18. The FlA crystal structure (PDB ID: 5B6I) with two interacting C-terminal (hexameric interface) domains colored beige; the K237 and E247 residues are shown as sticks and colored orange and grey, respectively.

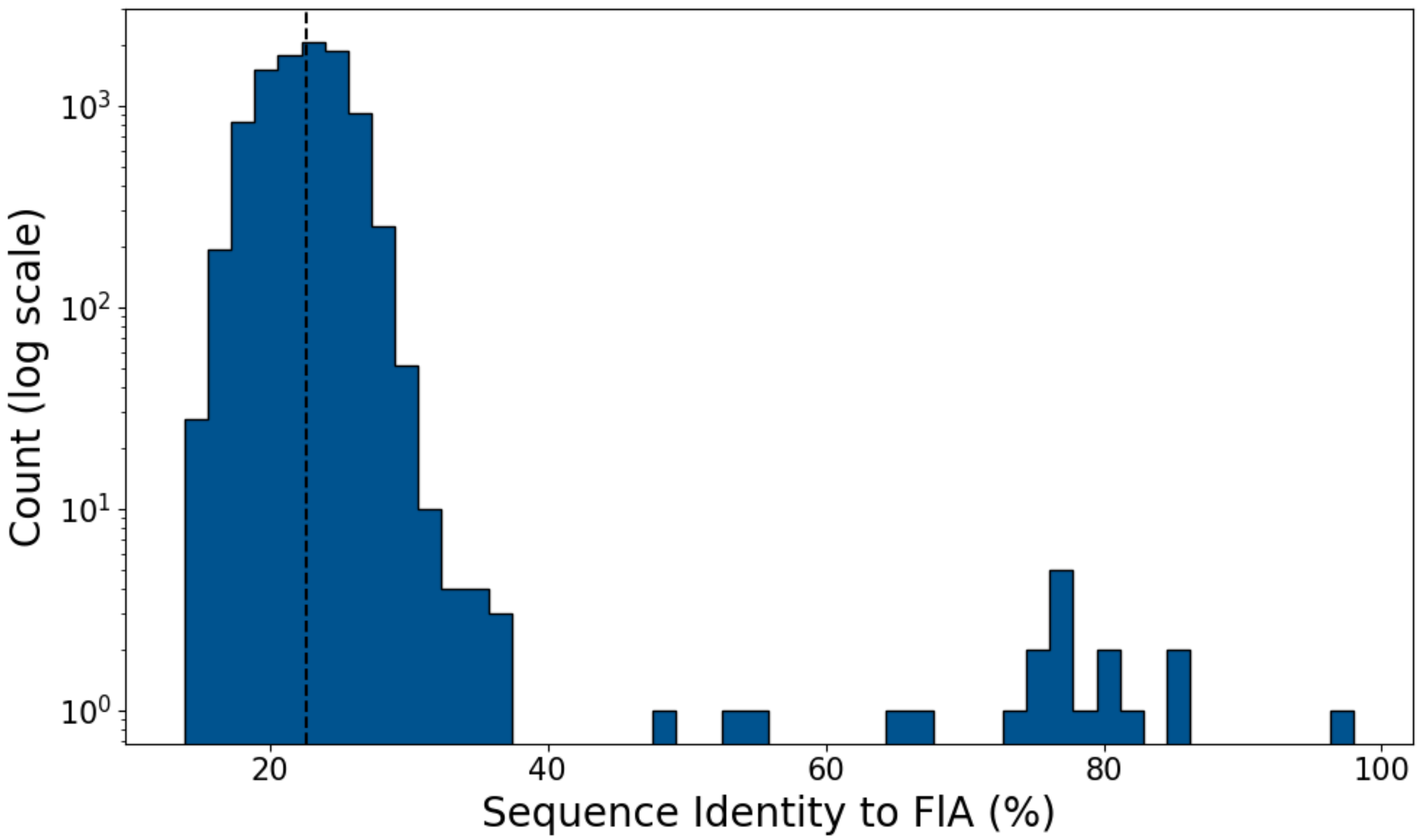

Figure S19. Distribution of sequence identities between FlA and all sequences in the MSA obtained from EVcouplings [1]. Identity was calculated over aligned positions excluding gaps, with the median identity, 22.6%, denoted with a dotted line.

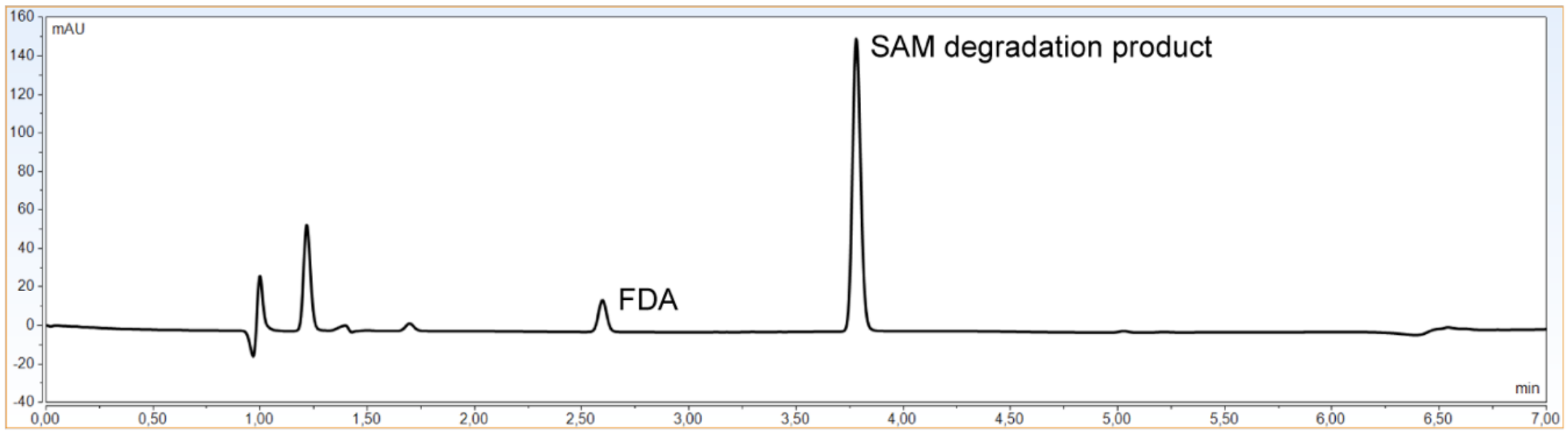

Figure S20. Representative HPLC trace showing FDA and SAM degradation product peaks at 260 nm.

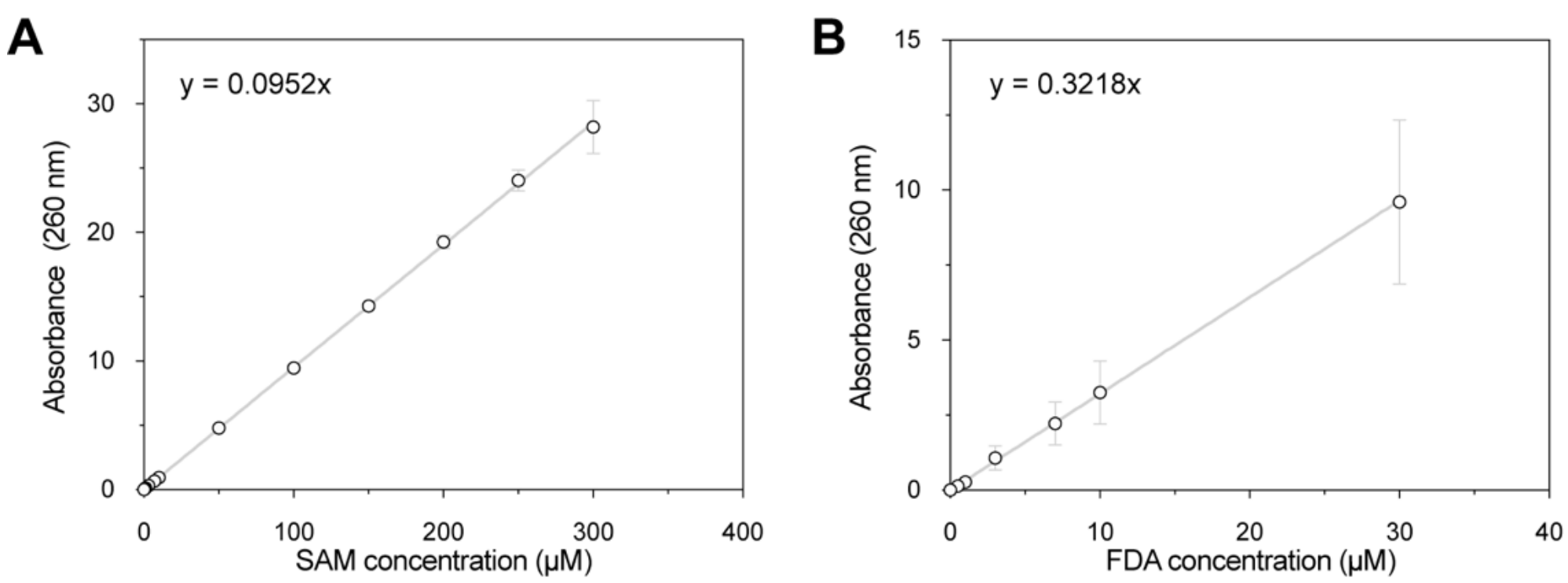

Figure S21. Calibration curves at A_{260} for **A** SAM (0–300 μM) and **B** FDA (0–30 μM). Points show mean absorbance, error bars indicate the standard deviation of biological replicates with N = 3, and lines indicate linear fits (slopes denoted on plots).

SUPPPORTING TABLES

Table S1. FIA variants from the original low-N dataset, with measured relative activity compared to wildtype (set at 100%).

Mutant ID	Mutations	Relative Activity (%)
HiM_001	E247G, R277L, Y286K	16.31
HiM_002	D241G, E247V	31.38
HiM_003	P285R, N287P	12.97
HiM_004	K237T, E247D, Y286P	4.60
HiM_005	P285V, Y286A, N287R	2.40
HiM_007	P285G, N287L	1.71
HiM_010	E247S	99.43
HiM_012	D241G, E247M, N287L	0.00
HiM_013	E247V, R277F, N287K	0.00
HiM_014	E247N, R277G, Y286N	0.00
HiM_015	E247D	9.39
HiM_016	E247Y, P285D	96.00
HiM_019	D241E, E247L, Y286I	0.00
HiM_020	Y286P, N287H	0.00
HiM_021	R277W, Y286S, N287K	0.00
HiM_027	R277F, N287L	0.00
HiM_029	P285L, Y286I	75.29
HiM_030	K237Q, D241E, R277F, P285Q, Y286I	0.00
HiM_031	N287T	48.85
HiM_032	K237M, D241I, P285S, N287A	0.00
HiM_033	K237I, E247I, R277V, Y286T, N287S	0.00
HiM_034	K237N, E247N, Y286L, N287T	1.72
HiM_035	N287Y	95.98
HiM_036	D241H, R277W, P285N	0.00
HiM_037	K237Y, R277M, P285S	18.39
HiM_038	K237L, E247R, Y286P, N287S	0.00
HiM_039	D241S, N287M	0.57
HiM_040	Y286L, N287V	5.75
HiM_041	D241V, E247V, R277S, Y286L, N287P	0.00
HiM_042	Y286V, N287D	3.84
HiM_043	K237Q, D241R, E247W, P285F, Y286A	0.00

HiM_044	D241S, N287H	80.40
HiM_045	P285R, N287T	94.89
HiM_046	E247H, R277F, N287K	87.22
HiM_047	E247R, R277F, Y286P, N287D	0.00
HiM_048	P285T, N287T	0.00
HiM_049	K237Y, R277L	1.14
HiM_050	E247A, R277I, P285I, Y286K	0.00
HiM_051	K237L, D241A, P285D, Y286C	1.70
HiM_052	D241S, E247S, R277L, P285H, Y286L	0.00

Table S2. FlA PRIZM variants, with measured relative activity compared to wildtype (set at 100%) and standard deviation of biological replicates (N = 3).

Category	Mutations[a]	ESM-1b Score	TranceptEVE Score	ESM-IF1 Score	Relative Activity 2 min (%)[b]	Relative Activity 180 min (%)[b]
ESM-1b Rankings						
1	Y77D	4.8793	-0.00093	-1.1027	0.55 ± 0.95	2.82 ± 2.76
2	Y77N	2.1799	-0.00474	-1.1108	1.48 ± 1.31	6.76 ± 1.14
3	P165N	1.7685	-0.00634	-1.1586	N/A	N/A
4	Y77E	1.6506	-0.00727	-1.1123	0.00 ± 0.00	0.00 ± 0.00
5	Y77H	1.4528	-0.00831	-1.1085	13.05 ± 2.06	49.93 ± 8.70
6	Y77S	1.1709	-0.00796	-1.1175	0.63 ± 1.10	4.45 ± 0.95
7	P165A	1.0006	-0.00177	-1.1392	51.94 ± 3.47	79.23 ± 2.92
8	Y77A	0.7652	-0.00832	-1.1178	3.29 ± 4.24	13.54 ± 11.67
9	P165T	0.6974	-0.00367	-1.1379	41.47 ± 8.52	71.50 ± 13.50
10	W103G	0.5558	-0.00566	-1.0960	50.70 ± 2.02	52.67 ± 3.95
TranceptEVE Rankings						
1	A298P	-4.3231	0.01243	-1.1305	117.22 ± 6.39	113.16 ± 4.69
2	E297T	-5.5349	0.01238	-1.1132	102.89 ± 6.58	99.23 ± 6.00
3	A3R	-4.0410	0.01211	-1.0952	106.65 ± 3.46	107.26 ± 1.44
4	R299I	-4.2130	0.01158	-1.1022	105.24 ± 2.93	106.59 ± 0.58
5	A2Q	-4.9021	0.01139	-1.1024	115.00 ± 8.76	111.27 ± 5.52
6	G5R	-3.4245	0.00844	-1.0934	105.80 ± 4.04	106.59 ± 2.09
7	**H32L**	-1.7019	0.00826	-1.0909	115.68 ± 7.67	109.61 ± 2.34
8	D108G	-2.7780	0.00766	-1.0999	103.20 ± 2.04	101.95 ± 1.81
9	F265A	-0.6425	0.00745	-1.1176	82.38 ± 2.55	103.37 ± 2.44
10	I263P	-2.8642	0.00577	-1.1258	2.52 ± 2.27	79.97 ± 5.21
ESM-IF1 Rankings						
1	F65V	-3.7128	-0.00864	-1.0732	51.51 ± 1.81	46.79 ± 2.22
2	F65I	-4.3913	-0.01101	-1.0755	N/A	N/A
3	F190I	-3.5711	-0.00275	-1.0772	87.09 ± 2.16	87.89 ± 4.19
4	F190L	-1.7038	-0.00226	-1.0785	94.11 ± 10.41	102.07 ± 9.39
5	S158G	-1.1291	-0.00022	-1.0818	1.03 ± 1.79	0.92 ± 0.055
6	**G37D**	-2.0180	0.00329	-1.0836	112.97 ± 0.07	104.40 ± 2.35
7	F65L	-3.4303	-0.00709	-1.0840	79.30 ± 1.77	74.91 ± 1.90
8	T76V	0.27275	-0.00013	-1.0841	26.69 ± 6.39	35.61 ± 6.26
9	F190V	-4.2351	-0.00518	-1.0841	97.75 ± 2.05	97.33 ± 0.54
10	**S166A**	-0.0739	0.00254	-1.0842	108.04 ± 1.34	95.79 ± 2.78
ESM-1b & TranceptEVE						
Above WT	N262E	0.3646	0.00459	-1.1039	65.52 ± 4.31	97.45 ± 3.21
Above WT	Y284E	0.1644	0.00209	-1.0987	42.14 ± 0.99	91.69 ± 3.59
Above WT	P285R	0.2337	0.00138	-1.1167	104.09 ± 3.27	106.73 ± 1.76
Above WT	Y284A	0.4831	0.00137	-1.1042	43.02 ± 2.77	89.48 ± 2.95
Above WT	Y284K	0.0456	0.00007	-1.1063	40.45 ± 1.24	91.17 ± 1.84
ESM-1b & ESM-IF1						
Above WT	T76V	0.2727	-0.00013	-1.0841	26.69 ± 6.39	35.61 ± 6.26
Above WT	W103G	0.5558	-0.00566	-1.0960	50.70 ± 2.02	52.67 ± 3.95

TranceptEVE & ESM-IF1[c]						
1	**H191E**	-1.6765	0.00427	-1.0866	120.73 ± 8.59	107.05 ± 2.17
2	**G37D**	-2.0180	0.00329	-1.0836	112.97 ± 0.07	104.40 ± 2.35
3	**S166A**	-0.0739	0.00254	-1.0841	108.04 ± 1.34	95.79 ± 2.78
4	H191D	-1.4522	0.00545	-1.0903	114.36 ± 10.90	104.92 ± 0.77
5	**H32L**	-1.7018	0.00826	-1.0909	115.68 ± 7.67	109.61 ± 2.34
6	H235R	-1.2666	0.00576	-1.0911	109.93 ± 11.60	110.53 ± 8.18
7	W50Q	-6.3206	0.00166	-1.0876	0.00 ± 0.00	0.62 ± 0.02
8	I239T	-2.3963	0.00516	-1.0916	116.34 ± 6.68	108.59 ± 2.04
9	H191K	-2.9410	0.00385	-1.0912	118.70 ± 4.06	110.01 ± 2.59
10	K234T	-2.8850	0.00343	-1.0910	109.86 ± 4.47	103.93 ± 2.53

[a] Bold names denote variants chosen for combinatorial analysis. [b] N/A: variants unable to express.
[c] Ranking based on combined ranking of the two models.

Table S3. MSA of fluorinase homologues[2] colored by PRIZM predictions[a]. Only positions with PRIZM predictions shown.

ResID[b]	MA37	PtaU1[c]	SAJ15[c]	Sxin[c]	Stro	Tnor	Pbac	Cbac	Scat	Amza	N902	Nbra2	Nbra3	Nbra1	AN11	Abar	CA12	PRIZM[d]
2	A	R	T	S	-	-	N	T	A	M	-	T	T	T	A	-	-	S
3	A	D	S	A	-	-	K	S	A	S	M	T	T	T	S	M	M	R
5	G	G	G	P	-	M	V	P	S	S	A	N	N	N	R	T	K	QNTRK
17	L	L	L	L	V	L	L	L	L	L	L	L	L	L	L	L	L	F
26	Q	I	Q	Q	L	L	I	I	Q	Q	Q	Q	Q	Q	Q	Q	Q	I
32	H	L	L	H	Y	I	L	L	Y	M	L	L	L	L	L	L	L	L
37	G	D	D	G	A	E	E	D	D	D	G	D	D	D	D	D	D	ED
50	W	W	F	W	F	F	F	F	W	W	W	W	W	W	W	W	W	FQ
83	T	S	E	E	A	E	M	S	T	T	A	T	T	T	T	G	T	AS
92	R	-	R	K	-	K	K	R	K	K	K	A	A	A	K	K	K	K
106	S	-	S	S	-	P	A	A	S	S	S	S	S	S	S	S	S	A
108	D	-	A	G	-	G	F	E	A	E	A	A	A	A	A	A	A	PAE
110	F	-	F	F	-	M	I	I	F	F	F	F	F	F	F	F	F	IE
114	D	P	E	E	E	E	V	E	E	E	E	E	E	E	E	E	E	E
120	I	V	I	V	V	V	V	I	I	I	I	I	I	I	I	I	I	V
129	T	R	P	T	F	F	D	S	T	T	T	T	T	T	S	S	S	SLR
137	I	V	I	I	V	I	L	L	L	T	I	L	L	L	L	L	V	L
146	K	G	E	K	D	E	N	S	K	D	K	E	E	E	E	E	E	E
166	S	A	S	A	A	A	S	A	S	S	S	S	S	S	S	S	S	A
176	A	E	D	S	A	S	S	S	S	E	N	E	E	E	N	E	E	E
181	R	P	P	P	R	P	P	P	P	K	A	R	R	R	P	K	P	K
183	D	N	K	E	D	T	G	Q	E	Q	S	A	A	A	A	A	A	A
191	H	D	S	Q	P	K	K	D	N	T	A	E	E	E	T	D	E	RNPTSAKDE
200	E	G	T	D	G	N	S	G	E	G	G	N	T	H	G	G	G	SND
203	S	Q	T	T	R	R	V	A	V	S	S	L	V	V	V	V	V	T
216	I	I	I	V	V	V	V	V	V	I	L	V	V	V	I	V	V	V
232	Q	Y	Y	Y	E	Y	Y	Y	Y	Y	Y	Y	Y	Y	Y	Y	Y	AEY
234	K	T	R	K	L	S	S	T	A	T	T	T	T	T	T	T	T	T
235	H	N	Q	R	E	R	Q	R	R	N	Q	E	K	K	K	E	R	NLVSQETKR
237	K	R	R	K	K	T	K	R	R	K	R	R	R	R	K	T	R	R
239	I	V	T	I	E	V	V	T	T	V	L	T	T	T	V	T	T	VT
252	P	R	P	P	K	R	Q	P	P	P	P	P	P	P	P	P	P	R
262	N	K	N	G	Q	D	A	A	N	D	D	A	A	A	T	S	T	SA
292	L	M	L	L	D	M	M	M	M	M	L	I	I	I	I	I	I	M

[a] Magenta = residues native to FlA (denoted as MA37), light green = residues predicted by PRIZM to be better than WT, dark green = residues both predicted *and* experimentally shown to be faster than WT. [b] Residue indices are based on MA37. [c] Faster than MA37. [d] PRIZM mutants either predicted to be faster (black), shown to be faster (green), or shown to be slower (red) for the given position.

REFERENCES

(1) Pardo, I.; Bednar, D.; Calero, P.; Volke, D. C.; Damborský, J.; Nikel, P. I. A Nonconventional Archaeal Fluorinase Identified by in Silico Mining for Enhanced Fluorine Biocatalysis. *ACS Catal.* **2022**, *12* (11), 6570–6577. https://doi.org/10.1021/ACSCATAL.2C01184.
